# Synthesis and Biological Evaluation of Novel 6-Hydroxy-benzo[*d*][1,3]oxathiol-2-one Schiff Bases as Potential Anticancer Agents

**DOI:** 10.3390/molecules20021968

**Published:** 2015-01-27

**Authors:** Eliza de Lucas Chazin, Paola de Souza Sanches, Eric Brazil Lindgren, Walcimar Trindade Vellasco Júnior, Laine Celestino Pinto, Rommel Mario Rodríguez Burbano, Julliane Diniz Yoneda, Kátia Zaccur Leal, Claudia Regina Brandão Gomes, James Lewis Wardell, Solange Maria Silva Veloso Wardell, Raquel Carvalho Montenegro, Thatyana Rocha Alves Vasconcelos

**Affiliations:** 1 Programa de Pós-Graduação em Química, Instituto de Química, Universidade Federal Fluminense, Outeiro de São João Batista s/no, Centro, Niterói 24020-141, RJ, Brazil; E-Mails: elizachazin@gmail.com (E.L.C.); paolasanches@id.uff.br (P.S.S.); ericblmail@gmail.com (E.B.L.); walcimar@gmail.com (W.T.V.J.); kzl@vm.uff.br (K.Z.L.); 2 School of Chemistry, University of Nottingham, University Park, Nottingham NG7 2RD, UK; 3 Fundação Oswaldo Cruz, Instituto de Tecnologia em Fármacos-Farmanguinhos, Rua Sizenando Nabuco 100, Manguinhos, Rio de Janeiro 21041-250, RJ, Brazil; E-Mails: claudiabrandao@far.fiocruz.br (C.R.B.G.); j.wardell@abdn.ac.uk (J.L.W.); 4 Instituto de Ciências Biológicas, Universidade Federal do Pará, Av. Augusto Corrêa 01, Guamá, Belém 66075-110, PA, Brazil; E-Mails: lainecelestino@hotmail.com (L.C.P.); rommel@ufpa.br (R.M.R.B.); rcm.montenegro@gmail.com (R.C.M.); 5 Departamento de Química, Instituto de Ciências Exatas, Universidade Federal Fluminense, Rua Desembargador Ellis Hemydio Figueira 783, Aterrado, Volta Redonda 27213-415, RJ, Brazil; E-Mail: jullianeyoneda@yahoo.com.br; 6 Department of Chemistry, University of Aberdeen, Old Aberdeen AB 24 3UE, Scotland; 7 CHEMSOL, 1 Harcourt Road, Aberdeen AB15 5NY, Scotland; E-Mail: solangewardell@yahoo.co.uk

**Keywords:** 1,3-benzoxathiol-2-ones, Schiff bases, anticancer, X-ray diffraction, Lipinski’s rule of five

## Abstract

With the aim of discovering new anticancer agents, we have designed and synthesized novel 6-hydroxy-benzo[*d*][1,3]oxathiol-2-one Schiff bases. The synthesis started with the selective nitration at 5-position of 6-hydroxybenzo[*d*][1,3]oxathiol-2-one (**1**) leading to the nitro derivative **2**. The nitro group of **2** was reduced to give the amino intermediate **3**. Schiff bases **4a**–**r** were obtained from coupling reactions between **3** and various benzaldehydes and heteroaromatic aldehydes. All the new compounds were fully identified and characterized by NMR (^1^H and ^13^C) and specifically for **4q** by X-ray crystallography. The* in vitro* cytotoxicity of the compounds was evaluated against cancer cell lines (ACP-03, SKMEL-19 and HCT-116) by using MTT assay. Schiff bases **4b** and **4o** exhibited promising cytotoxicity against ACP-03 and SKMEL-19, respectively, with IC_50_ values lower than 5 μM. This class of compounds can be considered as a good starting point for the development of new lead molecules in the fight against cancer.

## 1. Introduction

Cancer still remains a threat to huamn health, figuring among the leading causes of death worldwide [1,2]. In 2012, cancer was responsible for 8.2 million deaths and it is expected that annual cases will rise from 14 million in 2012 to 22 within the next two decades [1,3]. In the last years, many efforts have been made to develop new strategies for finding effective ways of treating this disease, which include not only an increase in the understanding of the biological process involved in cancer survival but also the search for more selective and potent chemotherapeutic agents [4].

In this context, several heterocyclic systems with five-membered rings fused to a benzene nucleus play an important role on designing of new drugs, since they display an interesting and diversified pharmacological profile. In particular, 1,3-benzoxathiol-2-one and its derivatives have been reported as important pharmacophores that exhibit antibacterial, antimycotic, antioxidant, antitumor, and anti-inflammatory activities [5,6,7,8]. In 2011, our research group has published the first review article highlighting the main aspects of the chemistry and biological properties of 1,3-benzoxathiol-2-ones [5].

Schiff bases have also been explored for their varied biological activities including antibacterial, anticancer, antioxidant, antifungal, antiviral, antimalarial, anti-inflammatory, antiglycation, angiotensin-II receptor antagonist, antidepressant and anticonvulsant [9,10,11,12,13].

Continuing our efforts on the synthesis of pharmacologically important heterocycles [14,15,16,17,18,19,20,21,22,23], we have decided to synthesize novel Schiff bases containing the 1,3-benzoxathiol-2-one moiety, an important heterocycle that has been little explored to date, and investigate their potential anticancer activity against different human cancer cell lines.

## 2. Results and Discussion

### 2.1. Chemistry

**Scheme 1:**
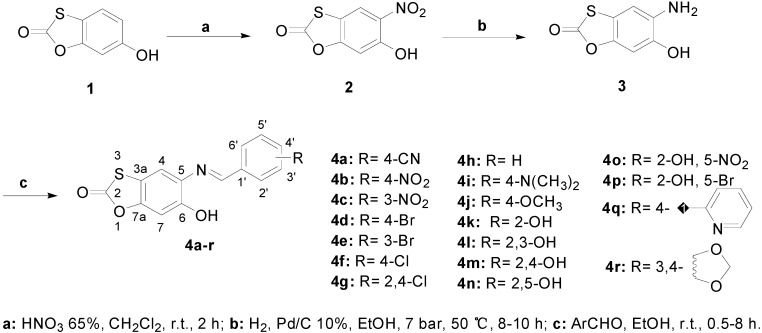
Synthesis of 1,3-benzoxathiol-2-one derivatives.

1,3-Benzoxathiol-2-one derivatives were prepared as shown in Scheme 1. The synthesis started with the selective nitration at 5-position of the commercially available 6-hydroxy-benzo[*d*][1,3]oxathiol-2-one (**1**) using HNO_3_ 65% and CH_2_Cl_2_ as solvent leading to the nitro derivative **2** in 75% yield. Melting point and spectral data (IR, ^1^H-NMR, ^13^C-NMR and ESI-MS) were consistent with literature data [24]. Key intermediate **3** was obtained in excellent yield (85%) by catalytic hydrogenation of **2** in ethanol with 10% Pd/C in a Berghof BR-300 reactor under 7 bar H_2_ pressure at 50 °C. It is noteworthy that this reaction was first attempted in ethanol at room temperature affording the product in very low yield, around 2%. Other classic methodologies like Fe/HCl; SnCl_2_/EtOH; Fe/NH_4_Cl 0.05 M have also been used, however none of these methods were successful in this case [25,26,27].

New compound **3** had its structure confirmed by spectral data (IR, ^1^H-NMR, ^13^C-NMR and ESI-MS). The IR spectrum indicated the reduction of the nitro group by the absence of the nitro vibrations at 1538 and 1275 cm^−1^ and by the presence of two N-H stretching vibrations at 3476 and 3389 cm^−1^. The ^1^H-NMR showed two singlets at 6.80 and 6.76 ppm for H7 and H4, respectively. The ^13^C-NMR spectrum exhibited the C=O signal at 170.5 ppm, carbons C6, C7a, C5 and C3a at 144.2, 138.9, 135.8 and 110.8 ppm, respectively and carbons C7 and C4 could be identified at 106.6 ppm and 99.2 ppm. Schiff bases **4a**–**r** were obtained from reactions of **3** with the appropriate benzaldehyde or heteroaromatic aldehyde in ethanol at room temperature (0.5–8 h) in 32%–82% yields. The structures of the new synthesized compounds were appropriately characterized by spectral data (^1^H-NMR, ^13^C-NMR, IR and ESI-MS). 2D-NMR techniques (COSY, HSQC and HMBC) helped us to assign the correct signals of the compounds. As an example, the ^1^H-NMR spectrum of compound **4b** exhibited two singlets at 9.86 and 8.87 ppm for O-H and imine proton (N=C-H), respectively. Protons H3'/H5' and H2'/H6' are shown as duplets at 8.36 ppm (*J* = 8.8 Hz) and 8.29 ppm (*J* = 8.8 Hz), respectively. Protons H7 and H4 appeared as singlets at 7.71 and 7.06 ppm. The ^13^C-NMR spectra exhibited the C=O signal at 169.7 ppm, C=N at 157.5 ppm and the C4' at 152.2 ppm. IR spectrum of compound **4b** showed the C=N stretching vibration at 1597 cm^−1^ and also the absence of the corresponding N-H vibrations.

### 2.2. Crystallography

**Figure 1:**
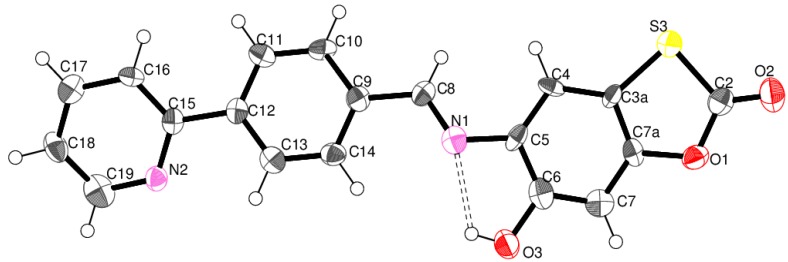
Atom arrangements and numbering scheme for **4q**.

Attempts were made to grow suitable crystals of various derivatives **4** for single crystal structure determinations. However, prolonged standing in alcohol solutions at room temperature generally led to deep colorations with deposited dark colored fine powders. Recrystallizations from other solvents were equally unsuccessful. Only far-from-ideal crystals of **4q**, grown from EtOH solution, were found to be of any use. However, while the data collected for these best crystals were disappointing, the atom connections within the molecule and the molecular conformation were securely established. While **4q** was not one of the active compounds, see Table 1, the conformation, particularly about the (benzylideneamino)-6-hydroxybenzo[*d*][1,3]oxathiol-2-one fragment is representative of those of the series **4**, as a whole. The structure of **4q**, deduced from the spectral data, was confirmed by the crystallographic study, including the (*E*)-stereochemistry about the C=N bond, see Figure 1 [28,29,30,31,32,33,34]. All bond lengths and angles are in the expected regions. The molecules of **4q** in the solid are very near planar.

**Table 1:** Cytotoxic activity of 1,3-benzoxathiol-2-one derivatives for cancer cell lines ^a^.

Compound	MTT			Hemolysis
	*IC_50_* μM			
	ACP-03	SKMEL-19	HCT-116	*EC*_50_ (μg/mL) ^b^
**2**	>10	>10	>10	>200
**3**	>10	>10	>10	>200
**4a**	>10	>10	>10	>200
**4b**	4.8 (3.2–7.2)	>10	>10	>200
**4c**	>10	>10	>10	>200
**4d**	>10	>10	>10	>200
**4e**	>10	>10	>10	>200
**4f**	>10	>10	>10	>200
**4g**	>10	>10	>10	>200
**4h**	>10	>10	>10	>200
**4i**	>10	>10	>10	>200
**4j**	>10	>10	>10	>200
**4k**	>10	>10	>10	>200
**4l**	>10	>10	>10	>200
**4m**	>10	9.4 (7.3–12.1)	>10	>200
**4n**	>10	5.6 (4.7–6.4)	>10	>200
**4o**	>10	2.8 (2.0–3.8)	>10	>200
**4p**	>10	>10	>10	>200
**4q**	>10	>10	>10	>200
**4r**	>10	>10	>10	>200
**Dox**	0.274 (0.22–0.33)	0.045 (0.01–0.15)	0.1 (0.05–0.28)	>200

Notes: ^a^ Data are presented as IC_50_ values and 95% of confidence interval for gastric (ACP-03), melanoma (SKMEL-19) and colon (HCT-116) cancer cells. Doxorubicin (Dox) was used as positive control. Experiments were performed in triplicate; ^b^ EC_50_ = effective concentration.

The hydroxyl group, O3–H3, is involved in a strong intramolecular hydrogen bond with the imine nitrogen (N): details are: O3–H3 = 0.84 Å, H3···N1 = 2.15 Å, O3···N = 2.633(8) Å and ∠O3–H3···N1 = 117°. A Platon analysis indicates another, but weaker, intramolecular C13–H13···N2 hydrogen bond. Several short intermolecular contacts of the types, C–H..N, C–H..O, C–H..π, C–O..π and π–π generate a three dimensional molecular array.

### 2.3. Molecular Modeling

**Figure 2:**
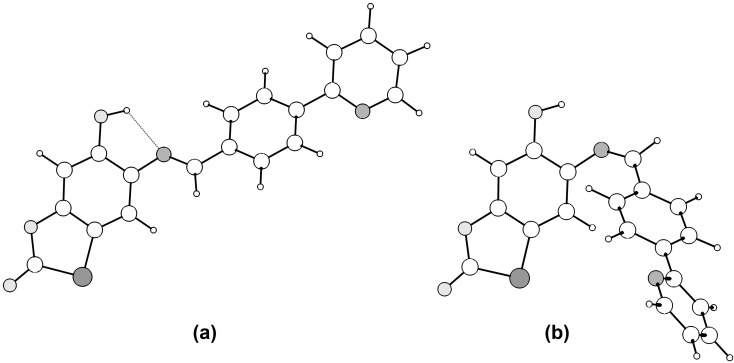
(**a**) *E* isomer, the lowest energy conformer and the only isomer observed experimentally; (**b**) *Z* isomer (Δ*G =* +4.83 kcal/mol).

The lowest energy conformer obtained corresponds to the *E* isomer found experimentally. For compound **4q**, its calculated structure is in agreement with the X-ray data (in parenthesis): O(6)–H(6) = 0.97 Å (0.84 Å), H(6)···N = 2.09 Å (2.15 Å), O(6)···N = 2.67 Å (2.633(8) Å) and ∠O(6)–H(6)···N = 116.6° (117°). A difference of 4.83 kcal/mol in the free energy (*G*) of isomers *E* and *Z* (Figure 2) was found. It should be noted the contribution of the stabilization effect provided by the formation of the strong intramolecular hydrogen bond between the imine nitrogen and the hydroxyl group for the *E* isomer, which is weaker in the *Z* isomer.

### 2.4. Biological Activity

*In vitro* anticancer activity of compounds **2**, **3** and **4a**–**r** was evaluated against three human cancer cell lines in comparison to doxorubicin by using MTT assay [35]. The antitumor activities are summarized in Table 1.

Results have shown that **4b** exhibited a good cytotoxicity against ACP-03 and compounds **4m**, **4n** and **4o** were considered to be active against SKMEL-19. Based on data collected from three independent experiments, compound **4o** was the most active with an IC_50_ value of 2.8 μM against melanoma cell line, whereas **4m** and **4n** have shown moderate activity for this same cancer cell line with IC_50_ values of 9.4 and 5.6 μM, respectively. Compound **4b** displayed a good cytotoxicity against ACP-03 with IC_50_ value of 4.8 μM. These results are in accordance to National Cancer Institute (NCI) protocols, where compounds exhibiting IC_50_ values <10 μM or 15 μM are considered active [36]. It is noteworthy that the active compounds bear nitro, hydroxyl or both groups in its structure, highlighting the most active compound **4o**, which bears a hydroxyl group at position 2' and a nitro group at position 5'. Activity reduction against SKMEL-19 was observed for dihydroxyl compounds **4m** and **4n** when compared to **4o**. This fact suggests the importance of these two different groups for the biological activity of this series. It is interesting to notice that the most active compound of a previously reported series of (*E*)-benzothiazole hydrazones against a specific leukemia (HL-60) cancer cell line follows this same pattern of substitution [17].

The mechanical stability of red blood cells is a good parameter for* in vitro* screening of unspecific cytotoxicity, since the membrane of erythrocyte can suffer significant changes in its structural properties [37]. In order to verify whether the cytotoxicity of the compounds was linked to the membrane disruption, the ability to induce lysis of mouse erythrocytes was investigated, and no membrane damage was found for all tested compounds (EC_50_ > 200 μg/mL). Therefore, we may suggest that the mechanism involved in cytotoxicity against cancer cell may not be related to nonspecific membrane damage (Table 1). In addition, none of these compounds exhibited cytotoxicity against the normal cells human fibroblast (MRC-5), murine fibroblast (NIH3T3) and normal human melanocyte (Melan-A).

In order to assess the potential oral bioavailability of all compounds, they were submitted to the Lipinski’s rule of five analysis (Table 2), which states that an orally active molecule should respect a molecular weight (MW) ≤ 500 g/mol, a partition coefficient octanol/water calculated clogP ≤ 5, a number of hydrogen bond acceptors (HBA) ≤ 10 and a number of hydrogen bond donors (HBD) ≤ 5 [38]. The related criteria Polar Surface Area (PSA) ≤ 140 A^2^, lately added by Veber* et al.* [39] was also included in the analysis.

All synthesized compounds fulfilled the parameters, which make them likely to be suitable for oral administration. The control compound, doxorubicin, failed to respect three of them: MW, PSA and HBA, which may explains its poor bioavailability after oral administration and why Dox chemotherapy is limited to intravenous administration or Dox-liposomes [40,41].

**Table 2:** Lipinski’s rule of five for compounds **2**, **3** and **4a**–**r**.

	M_W_ (Da)	PSA (A^2^)	HBA	HBD	ClogP
**2**	213.169	74.763	6	1	2.05
**3**	183.187	64.793	4	2	1.21
**4a**	296.31	60.46	5	1	3.91
**4b**	316.29	83.96	7	1	3.91
**4c**	316.29	83.92	7	1	3.91
**4d**	350.19	47.81	4	1	4.71
**4e**	350.19	45.05	4	1	4.71
**4f**	305.74	45.09	4	1	4.44
**4g**	340.19	43.30	4	1	5.00
**4h**	271.30	45.12	4	1	3.88
**4i**	314.37	45.92	5	1	4.16
**4j**	301.32	52.09	5	1	3.75
**4k**	287.30	60.01	5	2	3.49
**4l**	303.29	77.42	6	3	3.10
**4m**	303.29	78.77	6	3	3.10
**4n**	303.29	79.36	6	3	3.10
**4o**	332.29	98.31	8	2	3.52
**4p**	366.19	61.31	5	2	4.32
**4q**	348.38	51.36	5	1	4.64
**4r**	315.31	61.28	6	1	3.66
**Dox**	543.53	156.88	12	5	−0.68

## 3. Experimental Section

### 3.1. General Information

All reagents and solvents were used as obtained from commercial suppliers without further purification. Reactions were routinely monitored by thin-layer chromatography (TLC) on silica-gel precoated F_254_ Merck plates visualized under UV light (254–366 nm). Melting points were determined on a Fisatom 430 instrument (Fisatom, São Paulo, SP, Brazil) and are uncorrected. Catalytic hydrogenation reactions were performed on a Berghof BR-300 reactor (Berghof, Eningen, BW, Germany). IR spectra were recorded on a PerkinElmer 1420 spectrometer (PerkinElmer Inc., Waltham, MA, USA) using KBr pellets and frequencies are expressed in cm^−1^. Negative mode ESI-MS was done on a Waters ZQ-4000 single quadrupole mass spectrometer (Waters, Milford, MA, USA). NMR spectra were recorded on Varian Unity 300 and 500 (Varian Inc., Palo Alto, CA, USA) or on Bruker DRX 400 spectrometers (Bruker, Billerica, MA, USA) in DMSO-*d*_6_, CDCl_3_ or (CD3)_2_CO-*d*_6_. Chemical shifts (δ) are reported in ppm relative to tetramethylsilane. Elemental analysis was performed at CA IQ-USP, São Paulo, Brazil on a PerkinElmer-CHN 2400 analyzer (PerkinElmer Inc., Waltham, MA, USA).

### 3.2. Synthesis of 6-Hydroxy-5-nitrobenzo[d][1,3]oxathiol-2-one (**2**)

A solution of 6-hydroxy-benzo[*d*][1,3]oxathiol-2-one **1** (5 mmol) in CH_2_Cl_2_ (30 mL) was stirred for 30 min at 0 °C and then HNO_3_ 65% (10 mL) was added dropwise. The mixture was stirred for 2 h at room temperature and the acid excess was neutralized with a saturated aqueous solution of NaHCO_3_. The reaction mixture was extracted with CH_2_Cl_2_ (3 × 20 mL), the combined organic extracts were dried over MgSO_4_ and evaporated under reduced pressure to afford **2** as a yellow solid. Yield: 75%; m.p. 180–182 °C (m.p. lit. [24] 178–180 °C). IR (KBr, ν cm^−1^) 3216 (O-H); 1767 (C=O); 1538 (N-O); 1275 (N-O). ^1^H-NMR (CDCl_3_, 400.00 MHz, ppm): δ 10.90 (s, 1H, O-H); 8.22 (s, 1H, H4); 7.11 (s, 1H, H7). ^13^C-NMR (CDCl_3_, 100.0 MHz, ppm): δ 167.2 (C=O); 156.3 (C7a); 154.2 (C6); 131.6 (C5); 119.3 (C4); 115.8 (C3a); 103.2 (C7). ESI-MS: *m/z* [M−H]^−^: 212.1.

### 3.3. Synthesis of 5-Amino-6-hydroxybenzo[d][1,3]oxathiol-2-one (**3**)

To a mixture of 6-hydroxy-5-nitrobenzo[*d*][1,3]oxathiol-2-one (**2**) (4 mmol) and ethanol (150 mL) was added 10% Pd/C (220 mg). The catalytic hydrogenation was performed in a Berghof reactor under 7 bar H_2_ pressure at 50 °C. After 8–10 h, the catalyst was filtered off, washed with ethanol and evaporated under reduced pressure to yield **3** as a green solid. Yield: 85%; m.p. 194–195 °C. IR (KBr, ν cm^−1^) 3476 (N-H); 3389 (N-H); 1746 (C=O). ^1^H-NMR (DMSO-*d*_6_, 500.00 MHz, ppm): δ 6.80 (s, 1H, H7); 6.76 (s, 1H, H4). ^13^C-NMR (DMSO-*d*_6_, 125.0 MHz, ppm): δ 170.5 (C=O); 144.2 (C6); 138.9 (C7a); 135.8 (C5); 110.8 (C3a); 106.6 (C7); 99.2 (C4). ESI-MS: *m/z* [M−H]^−^: 182.1. Anal. Calcd. for C_7_H_5_NO_3_S: C, 45.90; H, 2.75; N, 7.65%, Found: C, 45.93; H, 2.62; N, 7.53%.

### 3.4. General Procedure for Synthesis of Schiff Bases **4a**–**r**

The Schiff bases **4a**–**r** were prepared from reactions between **3** (1 mmol) and the appropriate benzaldehyde or heteroaromatic aldehyde (1 mmol) in ethanol (10 mL). The system was kept under stirring at room temperature and the progress of the reactions was monitored by TLC using hexane/ethyl acetate (1:1) mixture as eluent. After reaction was completed (0.5–8 h), the solid product was collected by filtration and purified by washing with ethanol.

*(E)-4-((6-Hydroxy-2-oxobenzo[d][1,3]oxathiol-5-ylimino)methyl)benzonitrile* (**4a**). Yield: 82%; m.p. 261–263 °C. IR (KBr, ν cm^−1^) 3381 (O-H); 2224 (CN); 1739 (C=O); 1602 (C=N). ^1^H-NMR (DMSO-*d*_6_, 400.00 MHz, ppm): δ 9.82 (s, 1H, O-H); 8.81 (s, 1H, N=C-H); 8.22 (d, 2H, *J* = 8.4 Hz, H2'/H6'); 8.00 (d, 2H, *J* = 8.4 Hz, H3'/H5'); 7.70 (s, 1H, H7); 7.06 (s, 1H, H4). ^13^C-NMR (DMSO-*d*_6_, 125.0 MHz, ppm): δ 169.5 (C=O); 157.9 (N=C-H); 152.1 (C4'); 147.3 (C6 or C7a); 139.9 (C7a or C6); 135.2 (C5); 132.5 (C3'/C5'); 129.3 (C2'/C6'); 118.4 (C1'); 113.3 (C7); 113.0 (CN); 111.8 (C3a); 100.1 (C4). ESI-MS: *m/z* [M−H]^−^: 294.9.

*(E)-6-Hydroxy-5-(4-nitrobenzylideneamino)benzo[d][1,3]oxathiol-2-one* (**4b**). Yield: 81%; m.p. 223–224 °C. IR (KBr, ν cm^−1^) 3413 (O-H); 1747 (C=O); 1597 (C=N); 1515 (N-O); 1342 (N-O). ^1^H-NMR (DMSO-*d*_6_, 500.00 MHz, ppm): δ 9.86 (s, 1H, O-H); 8.87 (s, 1H, N=C-H); 8.36 (d, 2H, *J* = 8.8 Hz, H3'/H5'); 8.29 (d, 2H, *J* = 8.8 Hz, H2'/H6'); 7.71 (s, 1H, H7); 7.06 (s, 1H, H4). ^13^C-NMR (DMSO-*d*_6_, 125.0 MHz, ppm): δ 169.7 (C=O); 157.5 (N=C-H); 152.2 (C4'); 148.7 (C6 or C7a); 147.5 (C7a or C6); 141.6 (C1'); 135.2 (C5); 129.9 (C2'/C6'); 123.8 (C3'/C5'); 113.5 (C7); 111.9 (C3a); 100.4 (C4). ESI-MS: *m/z* [M−H]^−^: 314.9. Anal. Calcd. for C_14_H_8_N_2_O_5_S: C, 53.16; H, 2.55; N, 8.86%, Found: C, 52.73; H, 2.24; N, 8.86%.

*(E)-6-Hydroxy-5-(3-nitrobenzylideneamino)benzo[d][1,3]oxathiol-2-one* (**4c**). Yield: 73%; m.p. 220–222 °C. IR (KBr, ν cm^−1^) 3330 (O-H); 1748 (C=O); 1591 (C=N); 1530 (N-O); 1353 (N-O). ^1^H-NMR (DMSO-*d*_6_, 400.00 MHz, ppm): δ 9.90 (s, 1H, O-H); 8.89–8.87 (m, 2H, H_2_'/N=C-H); 8.43 (d, 1H, *J* = 7.6 Hz, H4'); 8.36 (dd, 1H, *^1^J* = 8.4; *^2^J* = 1.6 Hz, H6'); 7.82 (t, 1H, *J* = 7.6; H5'); 7.68 (s, 1H, H7); 7.06 (s, 1H, H4). ^13^C-NMR (DMSO-*d*_6_, 125.0 MHz, ppm): δ 169.5 (C=O); 157.6 (N=C-H); 152.0 (C3'); 148.0 (C6 or C7a); 147.2 (C7a or C6); 137.6 (C1'); 135.1 (C5); 135.0 (C6'); 130.2 (C4'); 125.4 (C2'); 122.8 (C5'); 113.4 (C7); 111.6 (C3a); 100.2 (C4). ESI-MS: *m/z* [M−H]^−^: 314.7.

*(E)-5-(4-Bromobenzylideneamino)-6-hydroxybenzo[d][1,3]oxathiol-2-one* (**4d**). Yield: 64%; m.p. 211–213 °C. IR (KBr, ν cm^−1^) 3362 (O-H); 1749 (C=O); 1622 (C=N). ^1^H-NMR (DMSO-*d*_6_, 500.00 MHz, ppm): δ 9.67 (s, 1H, O-H); 8.69 (s, 1H, N=C-H); 7.98 (d, 2H, *J* = 8.4 Hz, H2'/H6'); 7.73 (d, 2H, *J* = 8.4 Hz, H3'/H5'); 7.63 (s, 1H, H7); 7.04 (s, 1H, H4). ^13^C-NMR (DMSO-*d*_6_, 125.0 MHz, ppm): δ 169.9 (C=O); 158.6 (N=C-H); 151.7 (C4'); 146.9 (C1'); 135.8 (C6 or C7a); 135.2 (C7a or C6); 131.8 (C3'/C5'); 130.8 (C2'/C6'); 125.1 (C5); 113.1 (C7); 111.6 (C3a); 100.2 (C4). ESI-MS: *m/z* [M−H]^−^: 347.8.

*(E)-5-(3-Bromobenzylideneamino)-6-hydroxybenzo[d][1,3]oxathiol-2-one* (**4e**). Yield: 46%; m.p. 179–180 °C. IR (KBr, ν cm^−1^) 3334 (O-H); 1745 (C=O); 1625 (C=N). ^1^H-NMR (DMSO-*d*_6_, 500.00 MHz, ppm): δ 9.70 (s, 1H, O-H); 8.70 (s, 1H, N=C-H); 8.34 (s, 1H, H2'); 7.95 (d, 1H, *J* = 8.0, H4'); 7.72 (d, 1H, *J* = 8.0; H6'); 7.65 (s, 1H, H7); 7.48 (t, 1H, *J* = 7.8; H5'); 7.05 (s, 1H, H4). ^13^C-NMR (DMSO-*d*_6_, 100.0 MHz, ppm): δ 169.6 (C=O); 158.1 (N=C-H); 151.0 (C6 or C7a); 147.1 (C7a or C6); 138.3 (C5); 135.4 (C1'); 133.9 (C4'); 130.8 (C2'); 130.7 (C5'); 128.5 (C6'); 122.2 (C3'); 113.1 (C7); 111.7 (C3a); 100.3 (C4). ESI-MS: *m/z* [M−H]^−^: 347.7.

*(E)-5-(4-Chlorobenzylideneamino)-6-hydroxybenzo[d][1,3]oxathiol-2-one* (**4f**). Yield: 60%; m.p. 210–212 °C. IR (KBr, ν cm^−1^) 3354 (O-H); 1747 (C=O); 1624 (C=N). ^1^H-NMR (DMSO-*d*_6_, 500.00 MHz, ppm): δ 9.66 (s, 1H, O-H); 8.70 (s, 1H, N=C-H); 8.05 (d, 2H, *J* = 8.5 Hz, H2'/H6'); 7.63 (s, 1H, H7); 7.59 (d, 2H, *J* = 8.5 Hz, H3'/H5'); 7.04 (s, 1H, H4). ^13^C-NMR (DMSO-*d*_6_, 100.0 MHz, ppm): δ 169.7 (C=O); 158.5 (N=C-H); 151.8 (C4'); 146.9 (C6 or C7a); 144.0 (C7a or C6); 135.8 (C5); 134.9 (C1'); 130.6 (C3'/C5'); 128.8 (C2'/C6'); 113.2 (C7); 111.7 (C3a); 100.2 (C4). ESI-MS: *m/z* [M−H]^−^: 303.8.

*(E)-5-(2,4-Dichlorobenzylideneamino)-6-hydroxybenzo[d][1,3]oxathiol-2-one* (**4g**). Yield: 82%; m.p. 221–223 °C. IR (KBr, ν cm^−1^) 3366 (O-H); 1738 (C=O); 1593 (C=N). ^1^H-NMR (DMSO-*d*_6_, 500.00 MHz, ppm): δ 9.86 (s, 1H, O-H); 8.97 (s, 1H, N=C-H); 8.42 (d, 1H, *J* = 8.5 Hz, H6'); 7.75 (d, 1H, *J* = 2.0 Hz, H3'); 7.72 (s, 1H, H7); 7.57 (dd, 1H, *^1^J* = 8.5; *^2^J* = 2.0; H5'); 7.04 (s, 1H, H4). ^13^C-NMR (DMSO-*d*_6_, 125.0 MHz, ppm): δ 169.6 (C=O); 154.1 (N=C-H); 151.9 (C6 or C7a); 147.2 (C7a or C6); 136.5 (C5); 135.6 (C1'); 135.4 (C6'); 131.8 (C4'); 130.2 (C2'); 129.3 (C5'); 127.7 (C3'); 113.8 (C7); 112.0 (C3a); 100.3 (C4). ESI-MS: *m/z* [M−H]^−^: 337.9.

*(E)-5-(Benzylideneamino)-6-hydroxybenzo[d][1,3]oxathiol-2-one* (**4h**). Yield: 52%; m.p. 232–233 °C. IR (KBr, ν cm^−1^) 3321 (O-H); 1720 (C=O); 1627 (C=N). ^1^H-NMR (DMSO-*d*_6_, 500.00 MHz, ppm): δ 9.61 (s, 1H, O-H); 8.69 (s, 1H, N=C-H); 8.02 (dd, 2H, *^1^J* = 7.5; *^2^J* = 1.9; H2' or H6'); 7.61 (s, 1H, H7); 6.96 (m, 3H, H3' or H4' or H5'); 7.04 (s, 1H, H4). ^13^C-NMR (DMSO-*d*_6_, 125.0 MHz, ppm): δ 169.7 (C=O); 160.0 (N=C-H); 151.6 (C6 or C7a); 146.7 (C7a or C6); 136.2 (C5); 136.0 (C1'); 131.5 (C4'); 129.0 (C2'/C6'); 128.8 (C3'/C5'); 113.1 (C7); 111.6 (C3a); 100.1 (C4). ESI-MS: *m/z* [M−H]^−^: 270.2.

*(E)-5-(4-(Dimethylamino)benzylideneamino)-6-hydroxybenzo[d][1,3]oxathiol-2-one* (**4i**). Yield: 64%; m.p. 199–201 °C. IR (KBr, ν cm^−1^) 3307 (O-H); 1762 (C=O); 1594 (C=N). ^1^H-NMR (DMSO-*d*_6_, 500.00 MHz, ppm): δ 9.30 (s, 1H, O-H); 8.47 (s, 1H, N=C-H); 7.83 (d, 2H, *J* = 8.9 Hz, H2'/H6'); 7.53 (s, 1H, H7); 7.00 (s, 1H, H4); 6.78 (d, 2H, *J* = 8.9 Hz, H3'/H5'); 3.02 (s, 6H, CH_3_). ^13^C-NMR ((CD_3_)_2_CO-*d*_6_, 75.0 MHz, ppm): δ 170.3 (C=O); 159.5 (N=C-H); 154.1 (C4'); 153.4 (C6 or C7a); 147.9 (C7a or C6); 136.9 (C5); 131.9 (C2'/C6'); 124.8 (C1'); 113.2 (C3a); 112.5 (C3'/C5'); 111.3 (C7); 100.0 (C4); 40.3 (CH_3_). ESI-MS: *m/z* [M−H]^−^: 313.1.

*(E)-6-Hydroxy-5-(4-methoxybenzylideneamino)benzo[d][1,3]oxathiol-2-one* (**4j**). Yield: 40%; m.p. 177–178 °C. IR (KBr, ν cm^−1^) 3322 (O-H); 1744 (C=O); 1596 (C=N). ^1^H-NMR (DMSO-*d*_6_, 500.00 MHz, ppm): δ 9.48 (s, 1H, O-H); 8.60 (s, 1H, N=C-H); 7.97 (d, 2H, *J* = 8.7 Hz, H2'/H6'); 7.55 (s, 1H, H7); 7.07 (d, 2H, *J* = 8.7 Hz, H3'/H5'); 7.02 (s, 1H, H4); 3.85 (s, 3H, CH_3_). ^13^C-NMR (DMSO-*d*_6_, 125.0 MHz, ppm): δ 169.9 (C=O); 162.2 (C4'); 159.3 (N=C-H); 151.5 (C6 or C7a); 146.3 (C7a or C6); 136.6 (C5); 130.9 (C2'/C6'); 129.0 (C1'); 114.3 (C3'/C5'); 112.9 (C7); 111.6 (C3a); 100.1 (C4); 55.6 (CH_3_). ESI-MS: *m/z* [M−H]^−^: 300.0.

*(E)-6-Hydroxy-5-(2-hydroxybenzylideneamino)benzo[d][1,3]oxathiol-2-one* (**4k**). Yield: 63%; m.p. 252–253 °C. IR (KBr, ν cm^−1^) 3348 (O-H); 1765 (C=O); 1628 (C=N). ^1^H-NMR (DMSO-*d*_6_, 500.00 MHz, ppm): δ 13.27 (s, 1H, O-H); 10.36 (s, 1H, O-H); 8.93 (s, 1H, N=C-H); 7.76 (s, 1H, H7); 7.62 (dd, 1H, *^1^J* = 7.7; *^2^J* = 1.6; H6’); 7.40 (m, 1H, H4'); 7.05 (s, 1H, H4); 6.96 (m, 2H, H3' or H5'). ^13^C-NMR (DMSO-*d*_6_, 125.0 MHz, ppm): δ 169.5 (C=O); 162.0 (N=C-H); 160.4 (C2'); 151.4 (C6 or C7a); 146.9 (C7a or C6); 133.8 (C5); 133.1 (C4'); 132.2 (C6'); 119.5 (C1'); 119.0 (C5'); 116.7 (C3'); 113.7 (C7); 112.0 (C3a); 100.6 (C4). ESI-MS: *m/z* [M−H]^−^: 286.1.

*(E)-5-(2,3-Dihydroxybenzylideneamino)-6-hydroxybenzo[d][1,3]oxathiol-2-one* (**4l**). Yield: 50%; m.p. 244–246 °C. IR (KBr, ν cm^−1^) 3348 (O-H); 1730 (C=O); 1631 (C=N). ^1^H-NMR (DMSO-*d*_6_, 500.00 MHz, ppm): δ 13.54 (s, 1H, O-H); 10.42 (s, 1H, O-H); 9.08 (s, 1H, O-H); 8.89 (s, 1H, N=C-H); 7.79 (s, 1H, H7); 7.04–7.05 (m, 2H, H4/H6'); 6.92 (d, 1H, *J* = 7.4; H4'); 6.77 (t, 1H, *J* = 7.4; H5'). ^13^C-NMR (DMSO-*d*_6_, 100.0 MHz, ppm): δ 169.6 (C=O); 161.9 (N=C-H); 151.4 (C2'); 149.9 (C3'); 146.8 (C6 or C7a); 145.8 (C7a or C6); 133.2 (C5); 122.4 (C6'); 119.3 (C5'); 118.6 (C1'); 118.5 (C4'); 113.5 (C7); 111.9 (C3a); 100.4 (C4). ESI-MS: *m/z* [M−H]^−^: 301.9.

*(E)-5-(2,4-Dihydroxybenzylideneamino)-6-hydroxybenzo[d][1,3]oxathiol-2-one* (**4m**). Yield: 40%; m.p. 284-285 °C (d). IR (KBr, ν cm^−1^) 3370 (O-H); 1726 (C=O); 1625 (C=N). ^1^H-NMR (DMSO-*d*_6_, 500.00 MHz, ppm): δ 13.67 (s, 1H, O-H); 10.23 (s, 1H, O-H); 10.18 (s, 1H, O-H); 8.74 (s, 1H, N=C-H); 7.68 (s, 1H, H7); 7.38 (d, 1H, *J* = 8.5 Hz, H6'); 7.01 (s, 1H, H4); 6.38 (dd, 1H, *^1^J* = 8.5; *^2^J* = 2.2; H5') 6.28 (d, 1H, *J* = 2.2 Hz, H3'). ^13^C-NMR (DMSO-*d*_6_, 125.0 MHz, ppm): δ 169.7 (C=O); 163.6 (C4'); 162.5 (C2'); 161.2 (N=C-H); 151.0 (C6 or C7a); 149.6 (C7a or C6); 134.1 (C6'); 133.9 (C5); 113.3 (C5'); 112.3 (C1'); 111.8 (C3a); 107.8 (C7); 102.5 (C3'); 100.4 (C4). ESI-MS: *m/z* [M−H]^−^: 301.9. Anal. Calcd. for C_14_H_9_NO_5_S: C, 55.44; H, 2.99; N, 4.62%, Found: C, 55.60; H, 2.86; N, 4.83%.

*(E)-5-(2,5-Dihydroxybenzylideneamino)-6-hydroxybenzo[d][1,3]oxathiol-2-one* (**4n**). Yield: 60%; m.p. 261–263 °C. IR (KBr, ν cm^−1^) 3301 (O-H); 1713 (C=O); 1612 (C=N). ^1^H-NMR (DMSO-*d*_6_, 500.00 MHz, ppm): δ 12.40 (s, 1H, O-H); 10.28 (s, 1H, O-H); 9.03 (s, 1H, O-H); 8.81 (s, 1H, N=C-H); 7.72 (s, 1H, H7); 7.03 (s, 1H, H4); 6.99 (d, 1H, *J* = 3.0; H6'); 6.85 (dd, 1H, *^1^J* = 8.8; *^2^J* = 3.0; H4'); 6.78 (d, 1H, *J* = 8.8; H3'). ^13^C-NMR (DMSO-*d*_6_, 100.0 MHz, ppm): δ 169.6 (C=O); 161.7 (N=C-H); 153.1 (C2'); 151.4 (C5'); 149.5 (C6 or C7a); 146.7 (C7a or C6); 134.1 (C5); 121.0 (C3'); 119.4 (C4'); 117.2 (C6'); 116.5 (C1'); 113.7 (C7); 111.8 (C3a); 100.4 (C4). ESI-MS: *m/z* [M−H]^−^: 301.9. Anal. Calcd. for C_14_H_9_NO_5_S: C, 55.44; H, 2.99; N, 4.62%, Found: C, 55.53; H, 2.74; N, 4.56%.

*(E)-6-Hydroxy-5-(2-hydroxy-5-nitrobenzylideneamino)benzo[d][1,3]oxathiol-2-one* (**4o**). Yield: 70%; m.p. 293–294 °C. IR (KBr, ν cm^−1^) 3070 (O-H); 1763 (C=O); 1615 (C=N); 1542 (N-O); 1300 (N-O). ^1^H-NMR (DMSO-*d*_6_, 400.00 MHz, ppm): δ 14.86 (s, 1H, O-H); 10.72 (s, 1H, O-H); 9.15 (s, 1H, N=C-H); 8.64 (d, 1H, *J* = 2.9; H6'); 8.23 (dd, 1H, *^1^J* = 9,3; *^2^J* = 2.9; H4'); 7.85 (s, 1H, H7); 7.06 (m, 2H, H3'/H4). ^13^C-NMR (DMSO-*d*_6_, 75.0 MHz, ppm): δ 169.2 (C=O); 168.1 (C2'); 159.1 (N=C-H); 151.4 (C5'); 147.4 (C6 or C7a); 138.4 (C7a or C6); 130.8 (C5); 128.3 (C4'); 128.1 (C6'); 118.7 (C1'); 118.1 (C3'); 113.2 (C7); 112.1 (C3a); 100.3 (C4). ESI-MS: *m/z* [M−H]^−^: 330.8. Anal. Calcd. for C_14_H_8_N_2_O_6_S: C, 50.60; H, 2.43; N, 8.43%, Found: C, 50.27; H, 2.16; N, 8.35%.

*(E)-5-(5-Bromo-2-hydroxybenzylideneamino)-6-hydroxybenzo[d][1,3]oxathiol-2-one* (**4p**). Yield: 58%; m.p. 250–252 °C. IR (KBr, ν cm^−1^) 3442 (O-H); 1728 (C=O); 1617 (C=N). ^1^H-NMR (DMSO-*d*_6_, 500.00 MHz, ppm): δ 13.15 (s, 1H, O-H); 10.37 (s, 1H, O-H); 8.92 (s, 1H, N=C-H); 7.88 (d, 1H, *J* = 2,5; H6'); 7.74 (s, 1H, H7); 7.53 (dd, 1H, *^1^J* = 8.8; *^2^J* = 2.5; H4'); 7.04 (s, 1H, H4); 6.93 (d, 1H, *J* = 8.8; H3').^13^C-NMR (DMSO-*d*_6_, 125.0 MHz, ppm): δ 170.0 (C=O); 160.2 (N=C-H); 159.8 (C2'); 152.1 (C6 or C7a); 147.6 (C7a or C6); 135.7 (C4'); 134.0 (C6'); 133.7 (C5); 121.9 (C1'); 119.5 (C3'); 114.0 (C7); 112.3 (C3a); 110.2 (C5'); 100.9 (C4). ESI-MS: *m/z* [M−H]^−^: 365.9.

*(E)-6-Hydroxy-5-(4-(pyridin-2-yl)benzylideneamino)benzo[d][1,3]oxathiol-2-one* (**4q**). Yield: 32%; m.p. 201–203 °C. IR (KBr, ν cm^−1^) 3397 (O-H); 1750 (C=O); 1618 (C=N). ^1^H-NMR (DMSO-*d*_6_, 500.00 MHz, ppm): δ 9.67 (s, 1H, O-H); 8.76 (s, 1H, N=C-H); 8.72 (d, 1H, *J* = 4.6 Hz, H3''); 8.25 (d, 2H, *J* = 8.3 Hz, H3'/H5'); 8.14 (d, 2H, *J* = 8.3 Hz, H2'/H6'); 8.07 (d, 1H, *J* = 8.0 Hz, H4''); 7.93 (td, 1H, *^1^J* = 8.0; *^2^J* = 1,8 Hz, H6''); 7.66 (s, 1H, H7); 7.40 (dd, 1H, *^1^J* = 8.0; *^2^J* = 4,6 Hz, H5''); 7.05 (s, 1H, H4). ^13^C-NMR (DMSO-*d*_6_, 75.0 MHz, ppm): δ 169.6 (C=O); 159.2 (N=C-H); 155.1 (C1''); 151.7 (C6 or C7a); 149.6 (C3''); 146.7 (C7a or C6); 141.2 (C4'); 137.2 (C6''); 136.4 (C5 or C1'); 136.1 (C1' or C5); 129.3 (C2'/C6'); 126.6 (C3'/C5'); 123.0 (C5''); 120.6 (C4''); 113.1 (C7); 111.6 (C3a); 100.1 (C4). ESI-MS: *m/z* [M−H]^−^: 347.1.

*(E)-5-(Benzo[d][1,3]dioxol-5-ylmethyleneamino)-6-hydroxybenzo[d][1,3]oxathiol-2-one* (**4r**). Yield: 40%; m.p. 232–233 °C. IR (KBr, ν cm^−1^) 3346 (O-H); 1752 (C=O); 1625 (C=N). ^1^H-NMR (DMSO-*d*_6_, 500.00 MHz, ppm): δ 9.45 (s, 1H, O-H); 8.59 (s, 1H, N=C-H); 7.77 (d, 1H, *J* = 1.4 Hz, H2'); 7.61 (s, 1H, H7); 7.42 (dd, 1H, ^1^*J* = 8.0; *^2^J* = 1.4 Hz, H6'); 7.05 (d, 1H, *J* = 8.0 Hz, H5'); 7.05 (s, 1H, H4); 6.12 (s, 2H, H1''). ^13^C-NMR (DMSO-*d*_6_, 100.0 MHz, ppm): δ 169.7 (C=O); 158.6 (N=C-H); 151.8 (C4'); 150.2 (C3'); 148.0 (C6 or C7a); 146.5 (C7a or C6); 135.8 (C5); 130.9 (C1'); 126.4 (C6'); 112.5 (C3a); 111.6 (C2'); 108.2 (C7); 106.7 (C5'); 101.7 (C1''); 100.0 (C4). ESI-MS: *m/z* [M−H]^−^: 314.1.

### 3.5. Cytotoxicity against Cancer Cell Lines

Compounds (0.312–20 μM) were tested for cytotoxic activity against three cancer cell lines: ACP-03 (gastric), SKMEL-19 (melanoma) and HCT-116 (colon). All cell lines were maintained in DMEM medium supplemented with 10% fetal bovine serum, 2 mM glutamine, 100 U/mL penicillin, and 100 μM streptomycin at 37 °C with 5% CO_2_. Each compound was dissolved in DMSO and diluted with water to obtain a concentration of 20 μM. They were incubated with the cells for 72 h. The negative control received the same amount of DMSO (0.005% in the highest concentration). Doxorrubicin was used as a positive control. The cell viability was determined by reduction of the yellow dye 3-(4,5-dimethyl-2-thiazol)-2,5-diphenyl-2*H*-tetrazolium bromide (MTT) to a blue formazan product after 48 h as described by Mosmann [35].

### 3.6. Cell Membrane Disruption

The test was performed in 96-well plates using a 2% mouse erythrocyte suspension in 0.85% NaCl containing 10 mM CaCl_2_. The compounds diluted as mentioned above were tested at concentrations ranging from 1.5 to 200 μg/mL. After incubation at room temperature for 30 min and centrifugation, the supernatant was removed and the liberated hemoglobin was measured spectrophotometrically at 540 nm. DMSO was used as a negative control and Triton X-100 (1%) was used as positive control. EC_50_ is the calculated effective dose that induced lysis on 50% that of the Triton X-100 [37].

### 3.7. Molecular Modeling

The lowest-energy conformer was obtained through the *equilibrium conformer* calculation of the Spartan’10 program [42] at the B3LYP/6-31G(d,p) level of theory. Thereafter, the obtained conformer (isomer *E*) and the isomer *Z* were fully optimized at the M06-2X/6-311++G(d,p) level of theory using the Gaussian 09W program [43]. Since no imaginary frequency was found, the optimized structures were characterized as minima.

## 4. Conclusions

A series of new 6-hydroxy-benzo[*d*][1,3]oxathiol-2-one derivatives has been synthesized in good yields from commercially available materials and screened for their* in vitro* anticancer activity against three cancer cell lines. Results pointed Schiff base **4o** as the most active for melanoma (SKMEL-19), suggesting the importance of the nitro and hydroxyl groups for the cytotoxicity. The absence of hemolytic effects indicates that the mechanism of cytotoxicity of the substances is not related with membrane disruption and is probably related to more specific pathways of the cells. These preliminary results encourage further investigation on 6-hydroxy-benzo[*d*][1,3]oxathiol-2-one derivatives aiming at the discovery of more potent antitumorals.

## Supplementary Materials

Supplementary materials can be accessed at: http://www.mdpi.com/1420-3049/20/02/1968/s1.
