# Supplementary material for: Synthesis and Biological Evaluation of Novel 6-Hydroxy-benzo[d][1,3]oxathiol-2-one Schiff Bases as Potential Anticancer Agents
Source: Molecules. 2015 Jan 27;20(2):1968–83. doi: 10.3390/molecules20021968 (PMC6272564; doi:10.3390/molecules20021968)
Supplement: Supplementary file 1 [file molecules-20-01968-s001.pdf]

Supplementary Materials

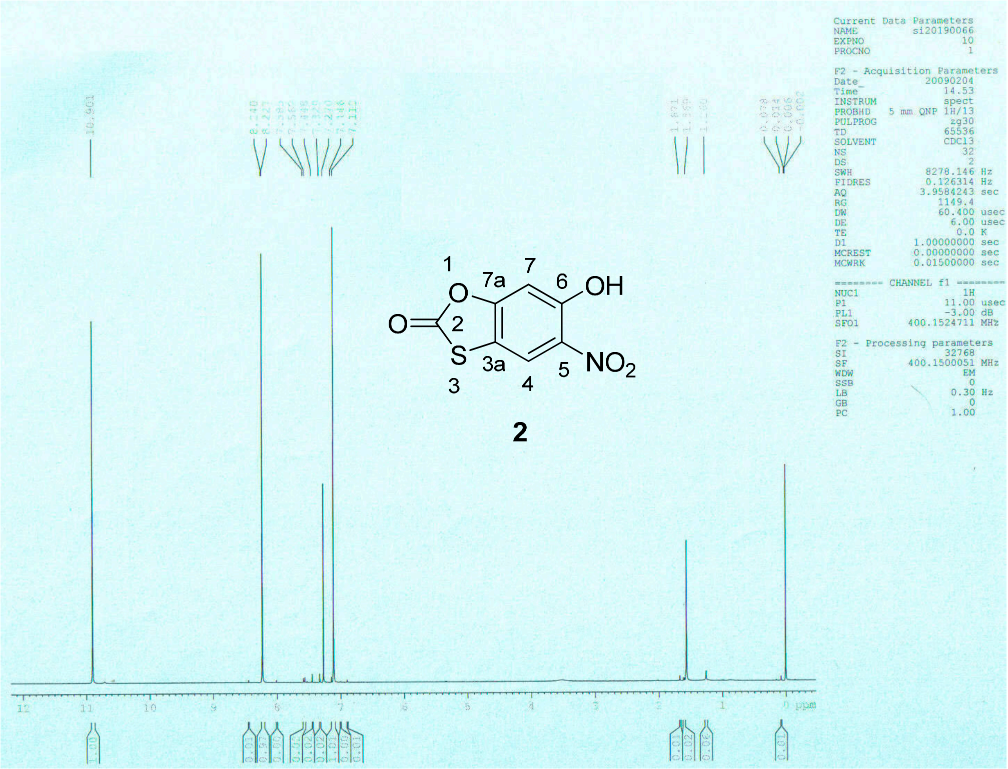

Figure S1. 6-Hydroxy-5-nitrobenzo[*d*][1,3]oxathiol-2-one (**2**).

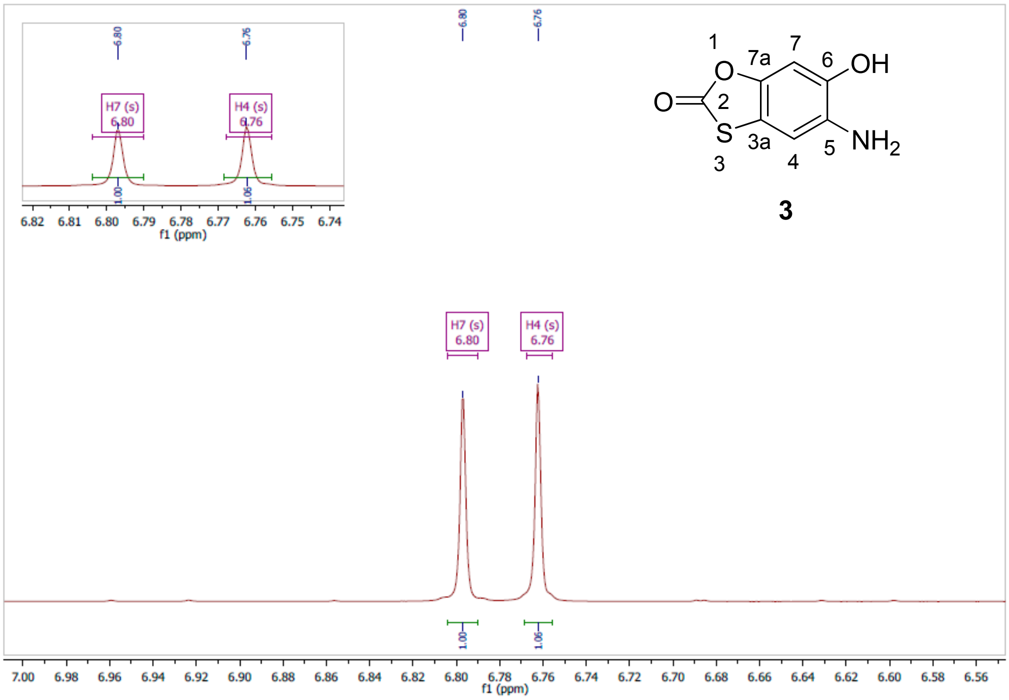

Figure S2. 5-Amino-6-hydroxybenzo[*d*][1,3]oxathiol-2-one (**3**).

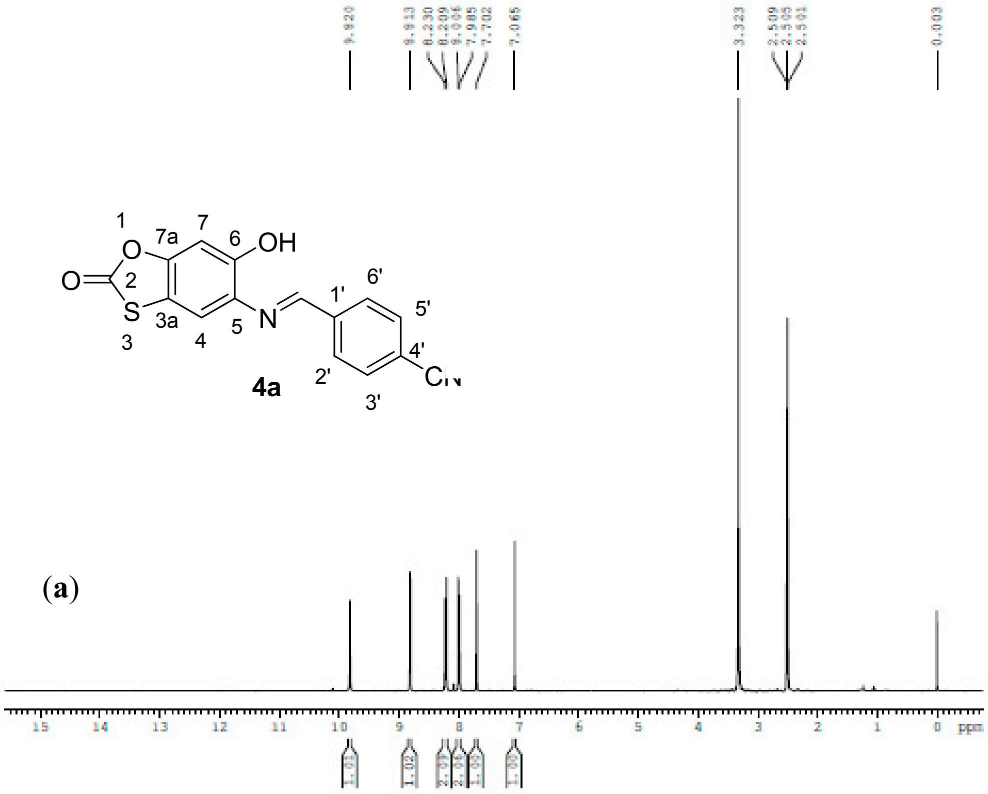

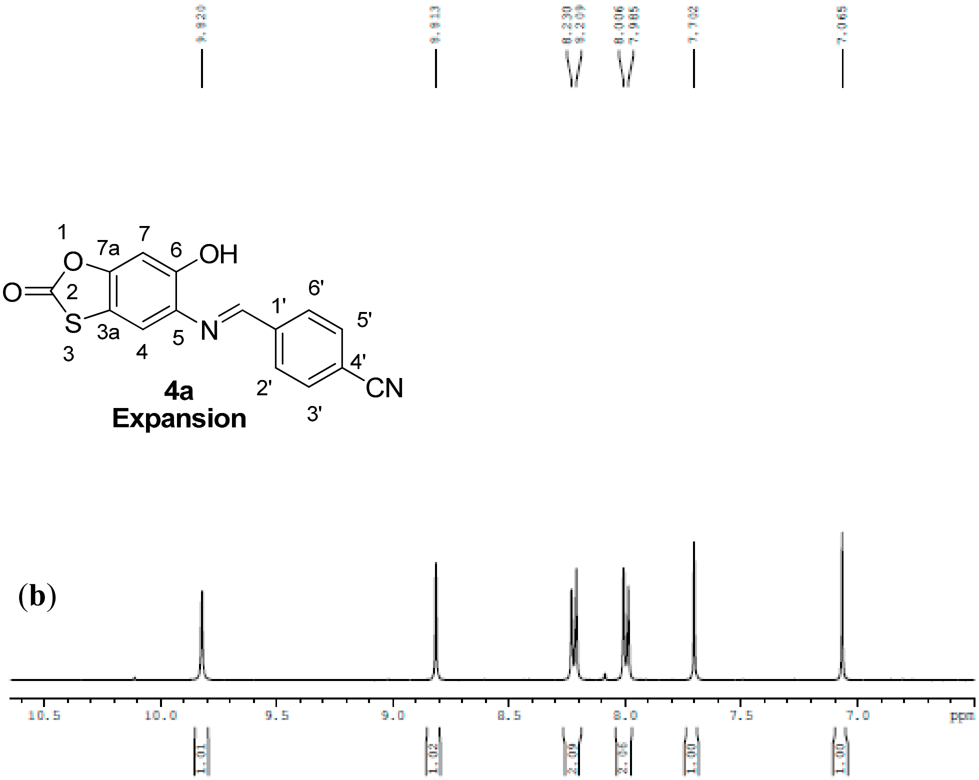

Figure S3. (**a**) (*E*)-4-((6-Hydroxy-2-oxobenzo[*d*][1,3]oxathiol-5ylimino)methyl)benzonitrile (**4a**), (**b**) Expansion.

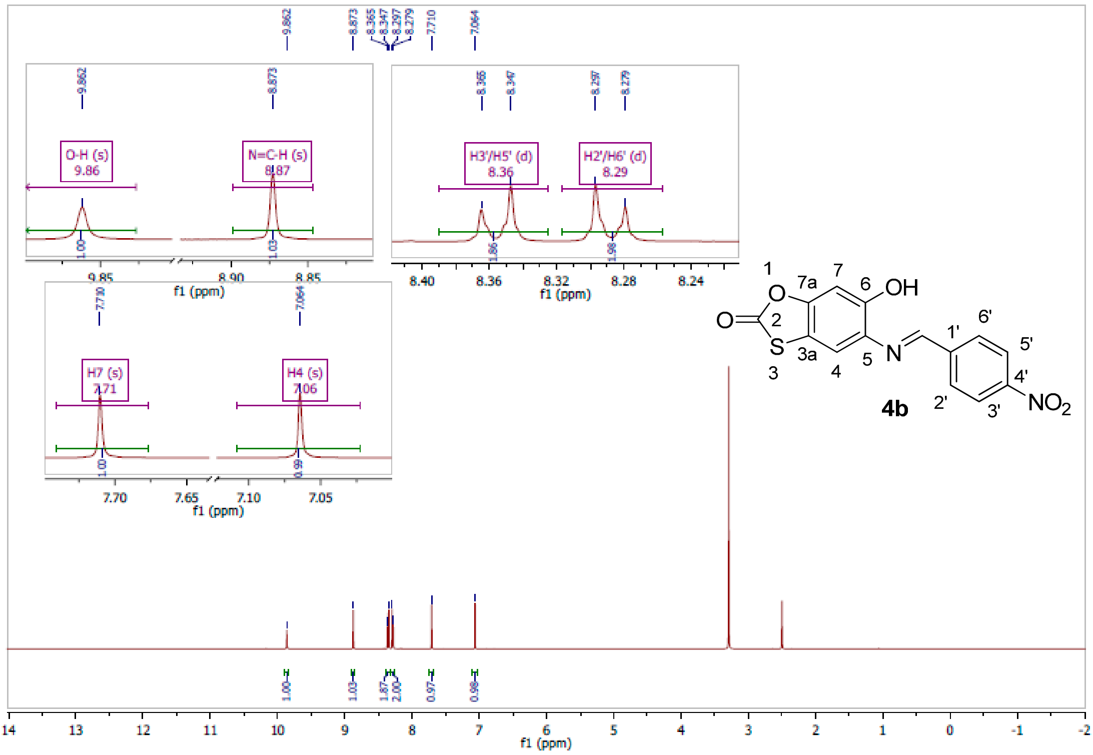

Figure S4. (*E*)-6-Hydroxy-5-(4-nitrobenzylideneamino)benzo[*d*][1,3]oxathiol-2-one (**4b**).

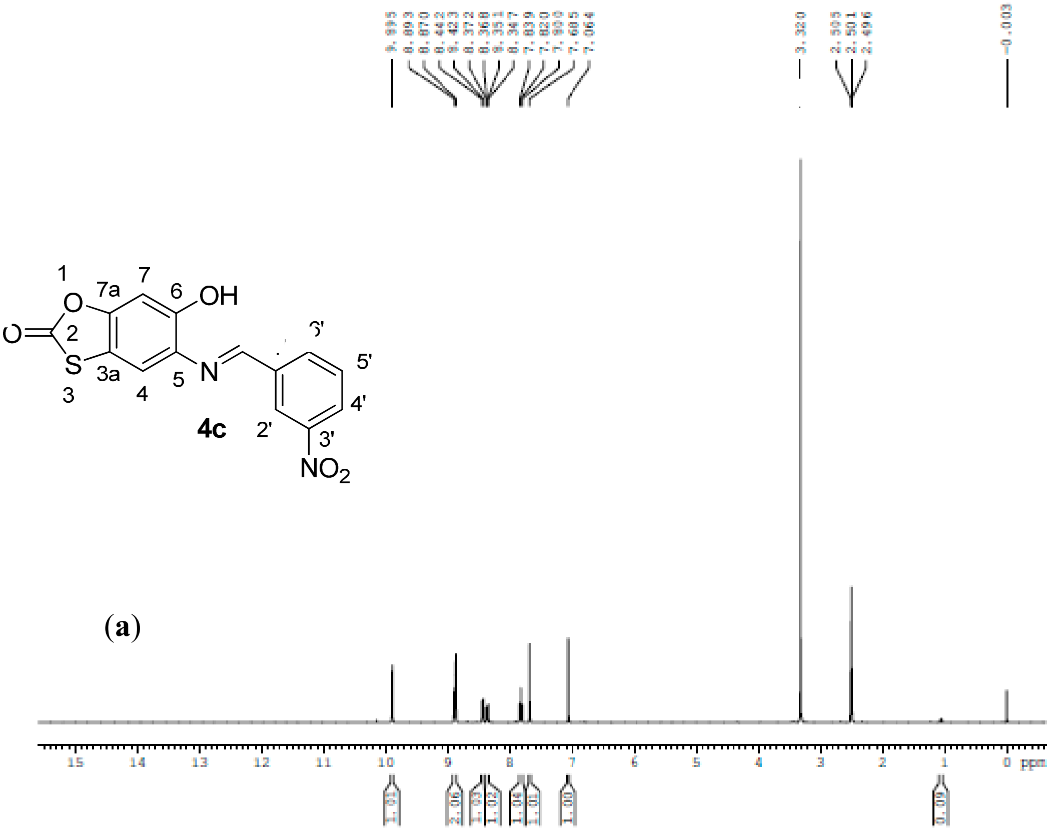

Figure S5. *Cont.*

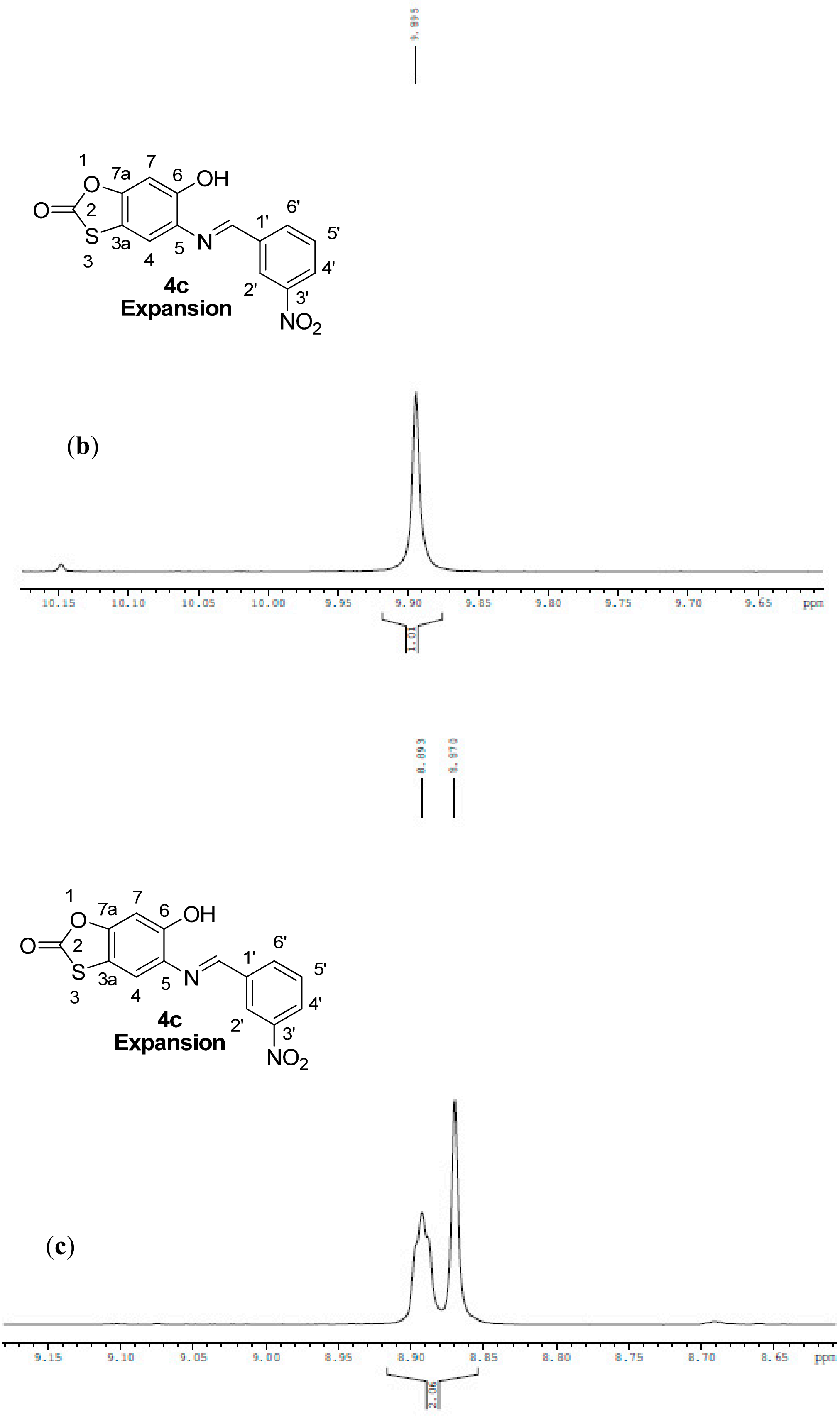

Figure S5. *Cont.*

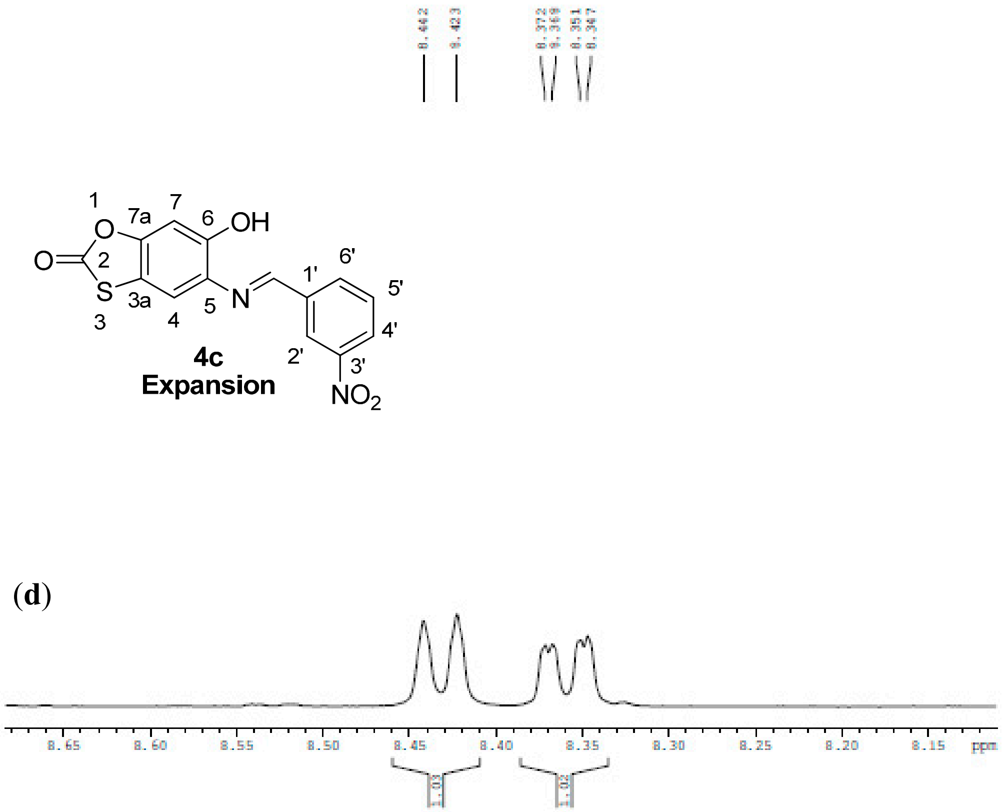

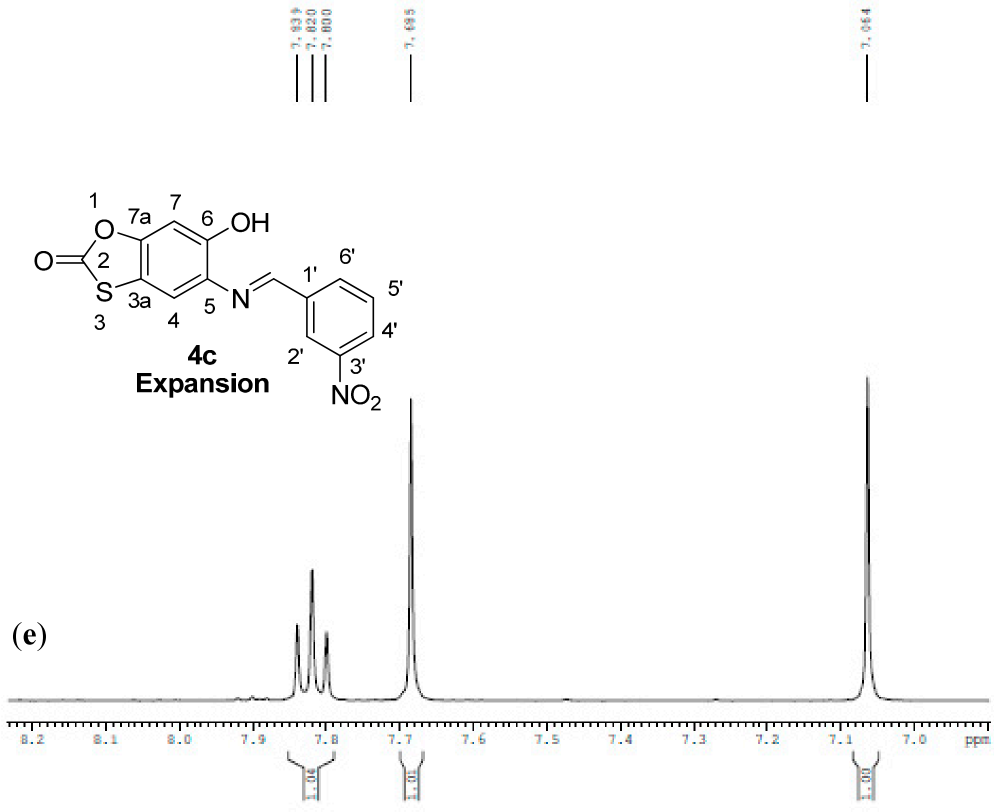

Figure S5. (**a**) (*E*)-6-Hydroxy-5-(3-nitrobenzylideneamino)benzo[*d*][1,3]oxathiol-2-one (**4c**). (**b**–**e**) Expansion.

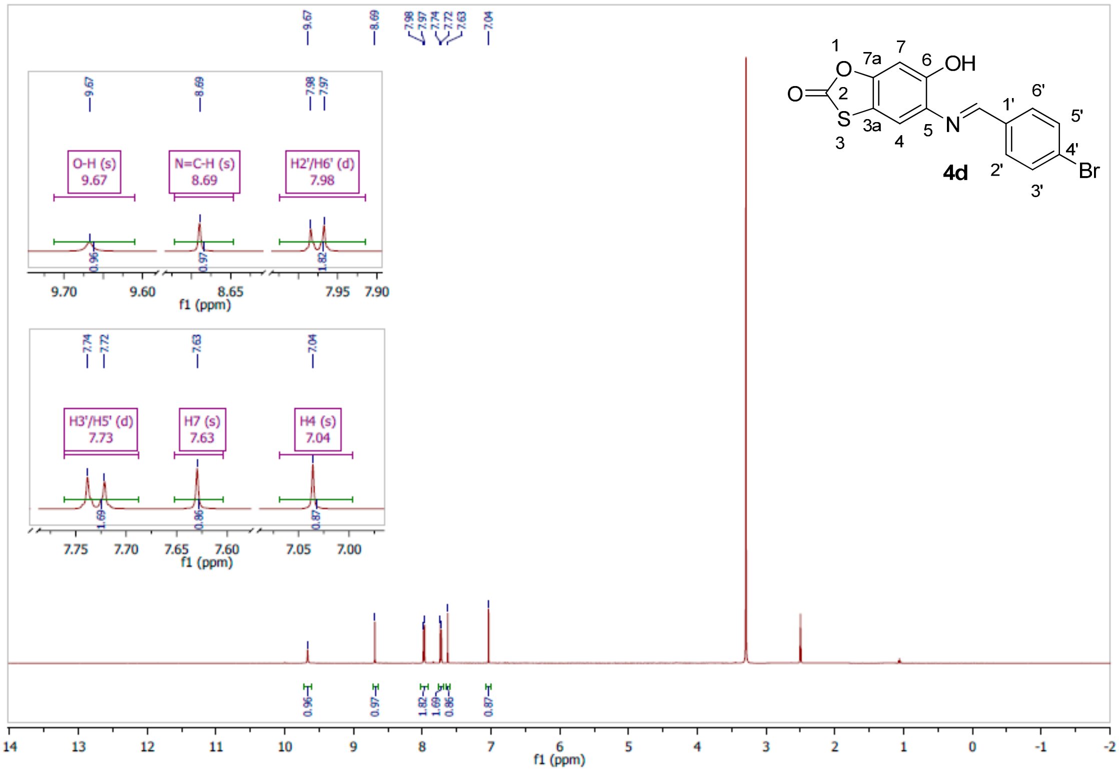

Figure S6. (*E*)-5-(4-Bromobenzylideneamino)-6-hydroxybenzo[*d*][1,3]oxathiol-2-one (**4d**).

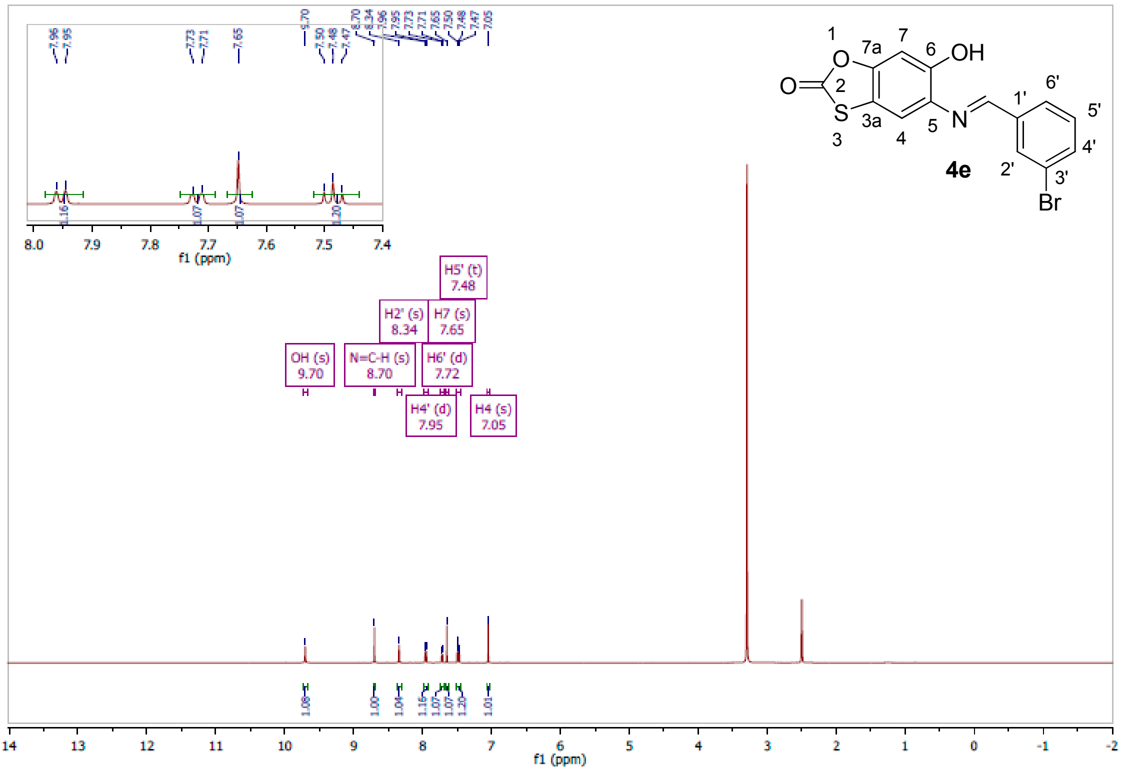

Figure S7. (*E*)-5-(3-Bromobenzylideneamino)-6-hydroxybenzo[*d*][1,3]oxathiol-2-one (**4e**).

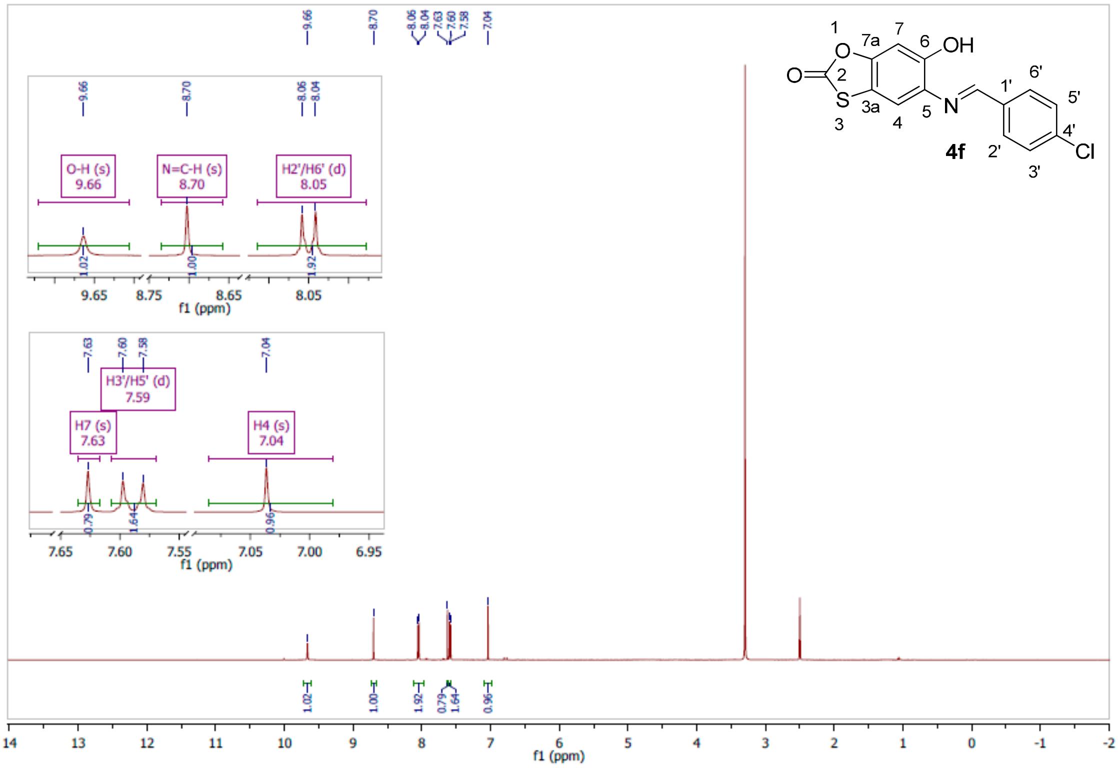

Figure S8. (*E*)-5-(4-Chlorobenzylideneamino)-6-hydroxybenzo[*d*][1,3]oxathiol-2-one (**4f**).

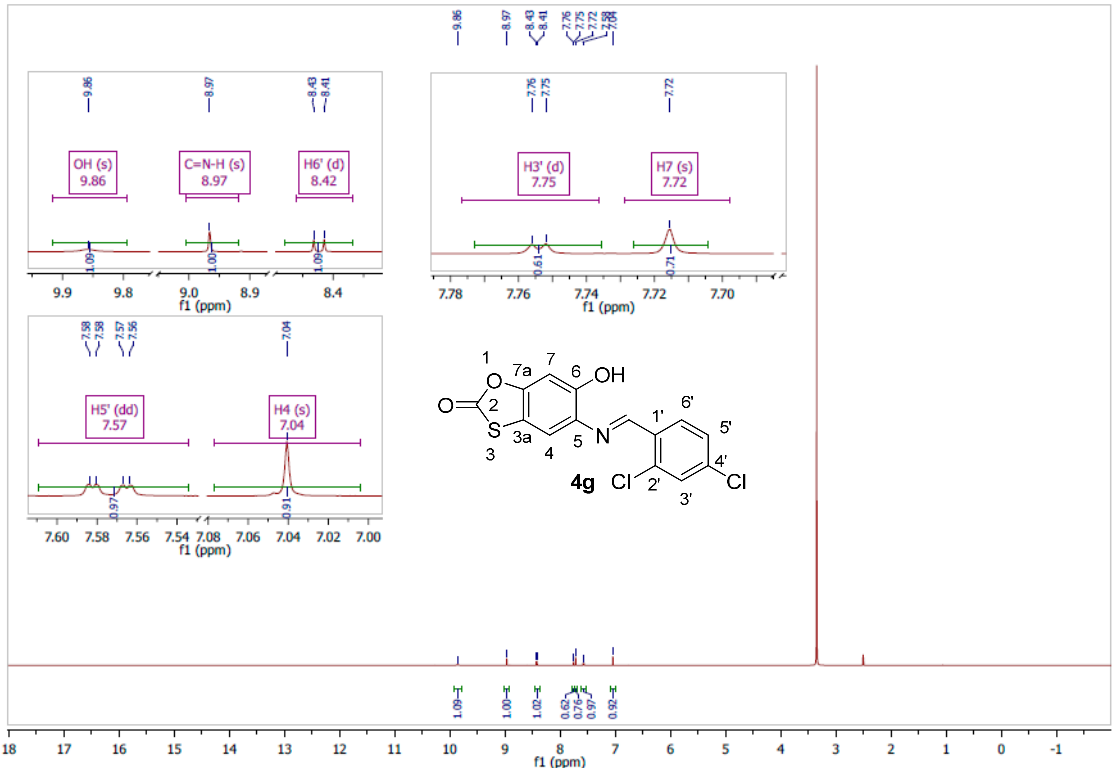

Figure S9. (*E*)-5-(2,4-Dichlorobenzylideneamino)-6-hydroxybenzo[*d*][1,3]oxathiol-2-one (**4g**).

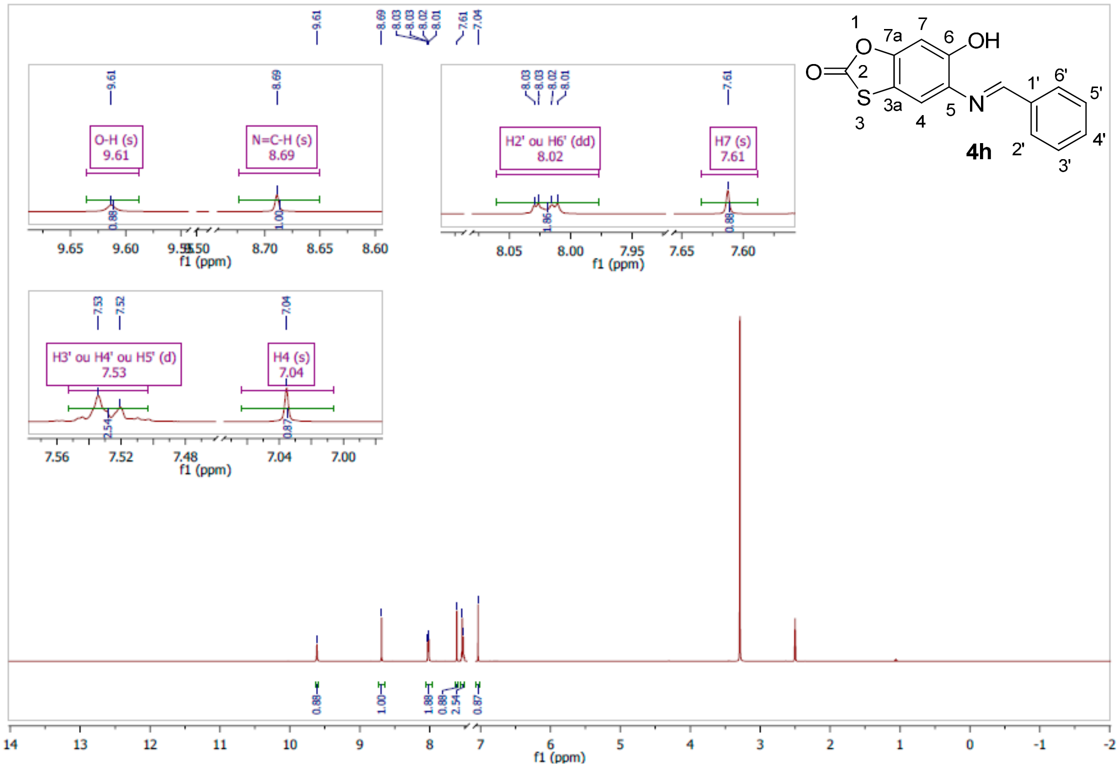

Figure S10. (*E*)-5-(Benzylideneamino)-6-hydroxybenzo[*d*][1,3]oxathiol-2-one (**4h**).

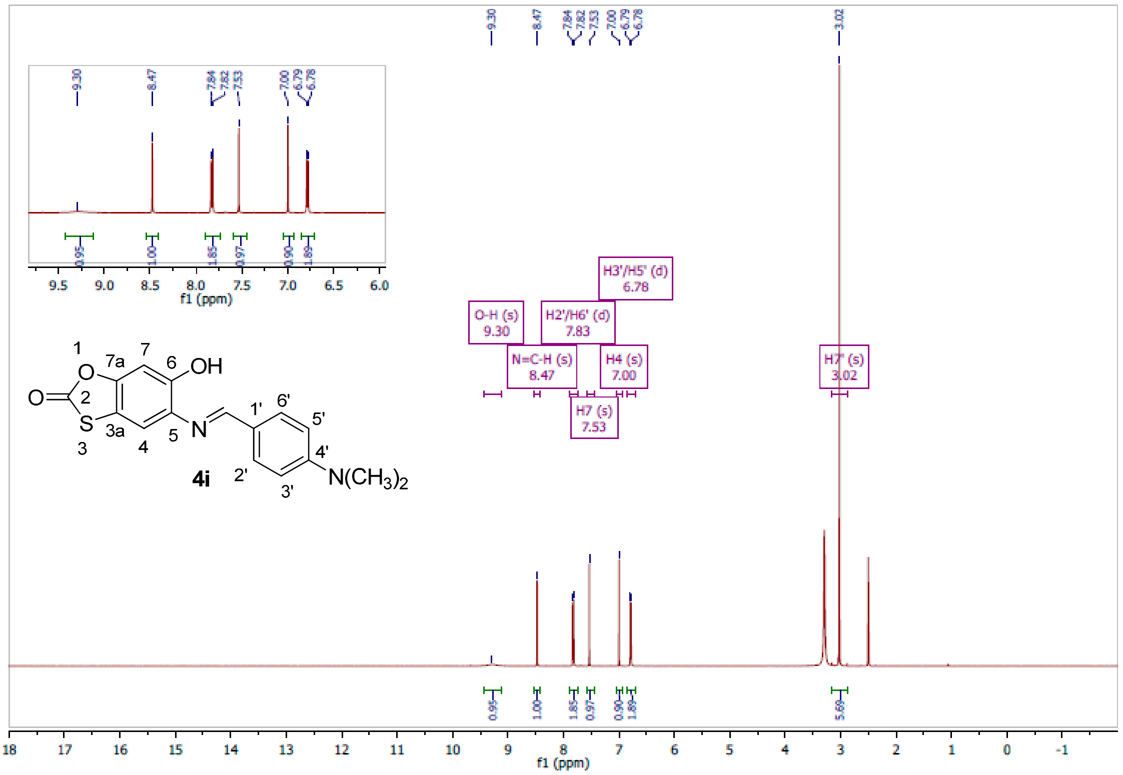

Figure S11. (*E*)-5-(4-(Dimethylamino)benzylideneamino)-6-hydroxybenzo[*d*][1,3]oxathiol-2-one (**4i**).

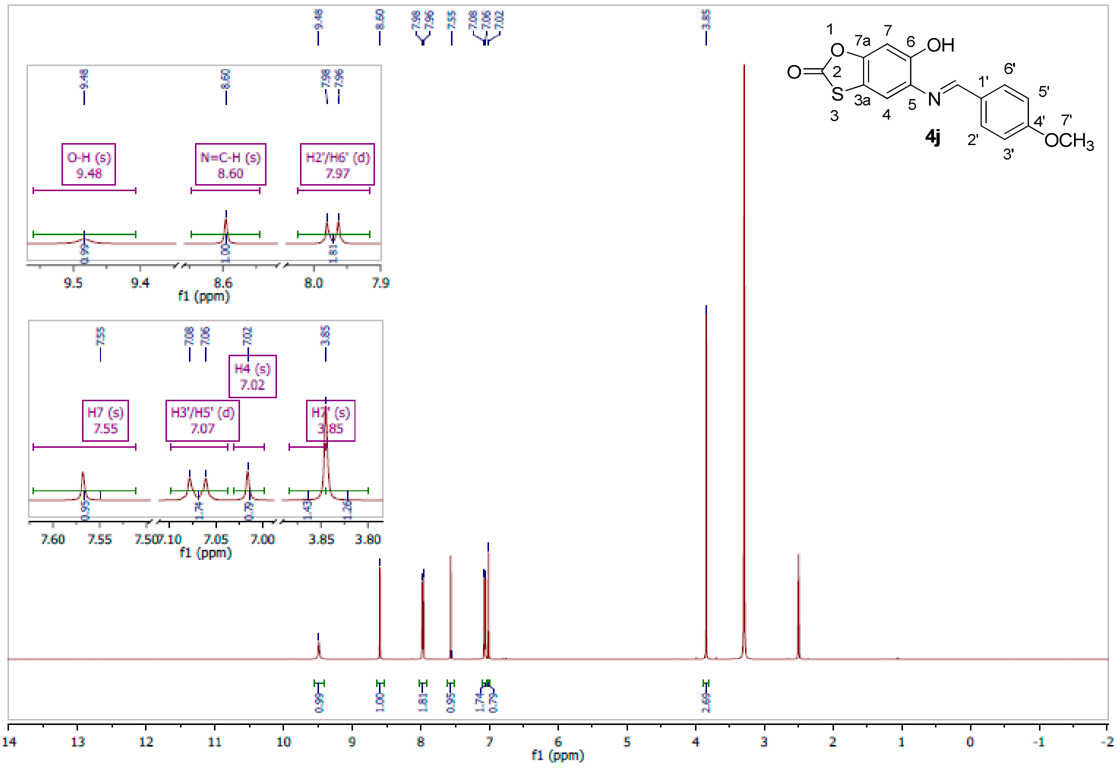

Figure S12. (*E*)-6-Hydroxy-5-(4-methoxybenzylideneamino)benzo[*d*][1,3]oxathiol-2-one (**4j**).

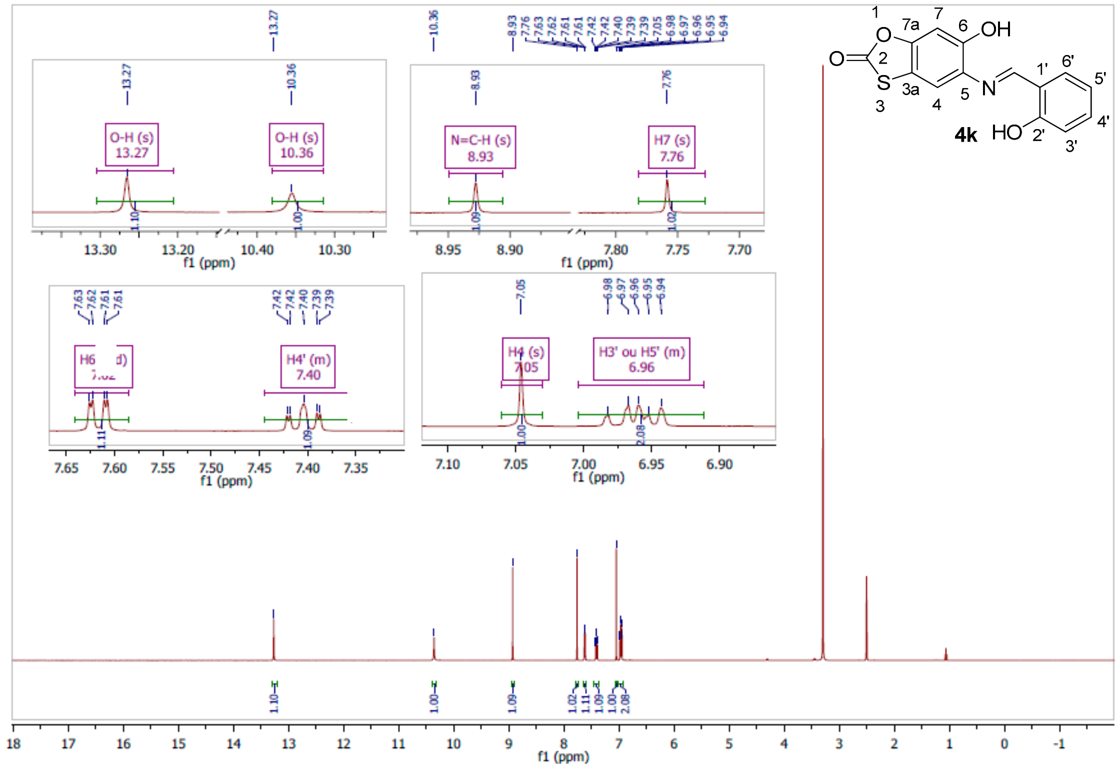

Figure S13. (*E*)-6-Hydroxy-5-(2-hydroxybenzylideneamino)benzo[*d*][1,3]oxathiol-2-one (**4k**).

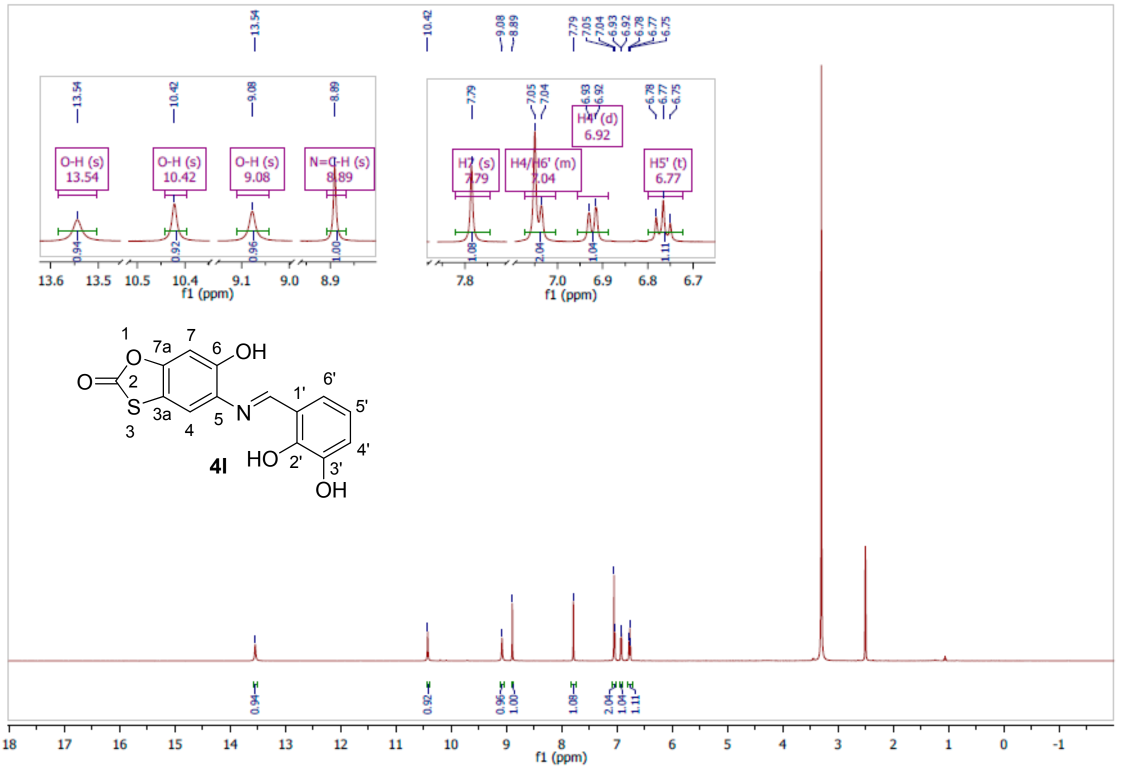

Figure S14. (*E*)-5-(2,3-Dihydroxybenzylideneamino)-6-hydroxybenzo[*d*][1,3]oxathiol-2-one (**4l**).

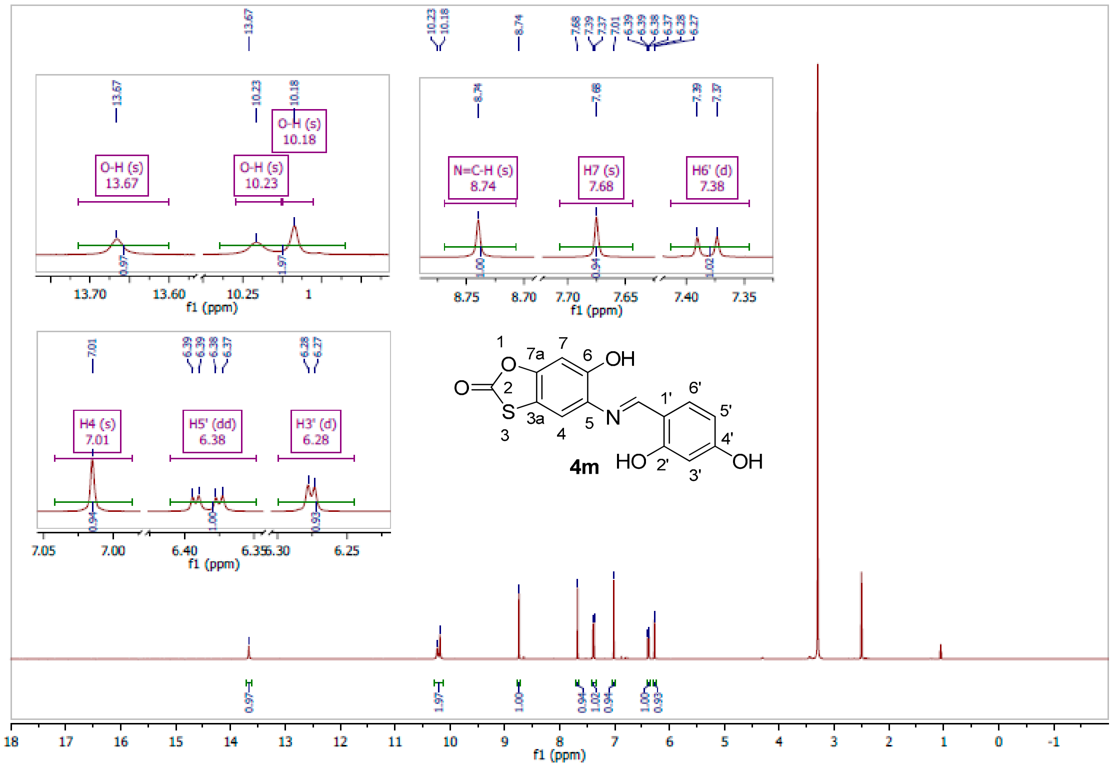

Figure S15. (*E*)-5-(2,4-Dihydroxybenzylideneamino)-6-hydroxybenzo[*d*][1,3]oxathiol-2-one (**4m**).

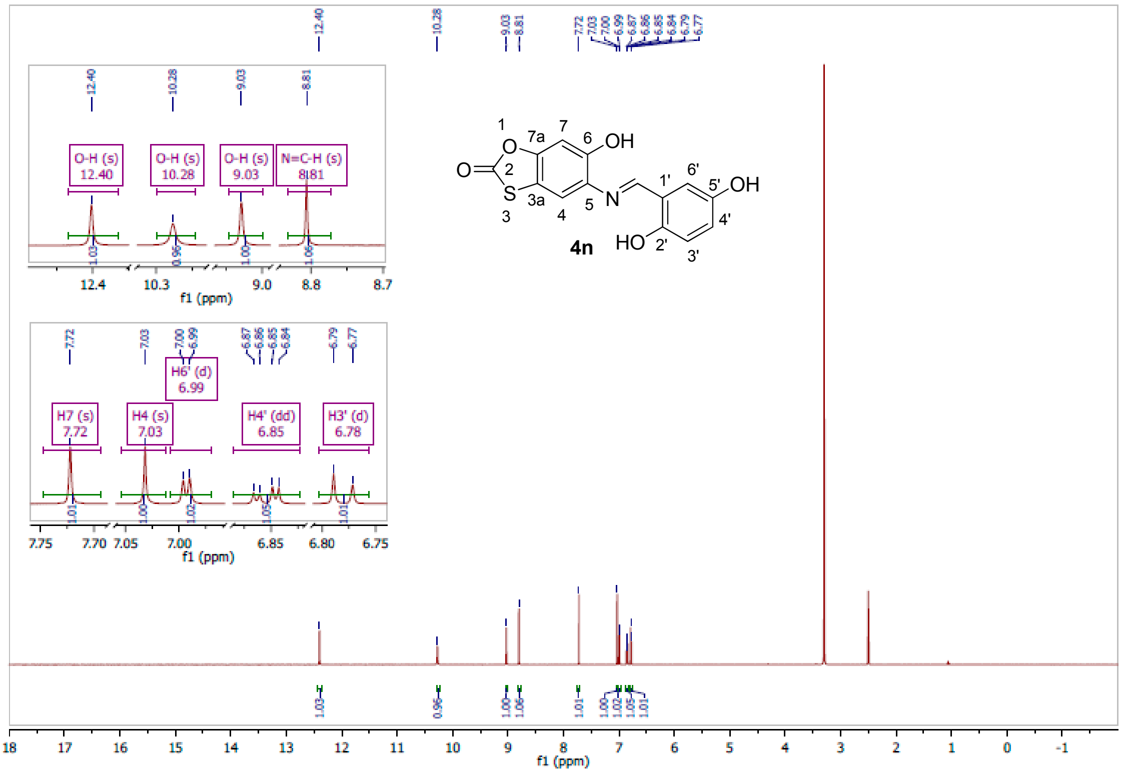

Figure S16. (*E*)-5-(2,5-Dihydroxybenzylideneamino)-6-hydroxybenzo[*d*][1,3]oxathiol-2-one (**4n**).

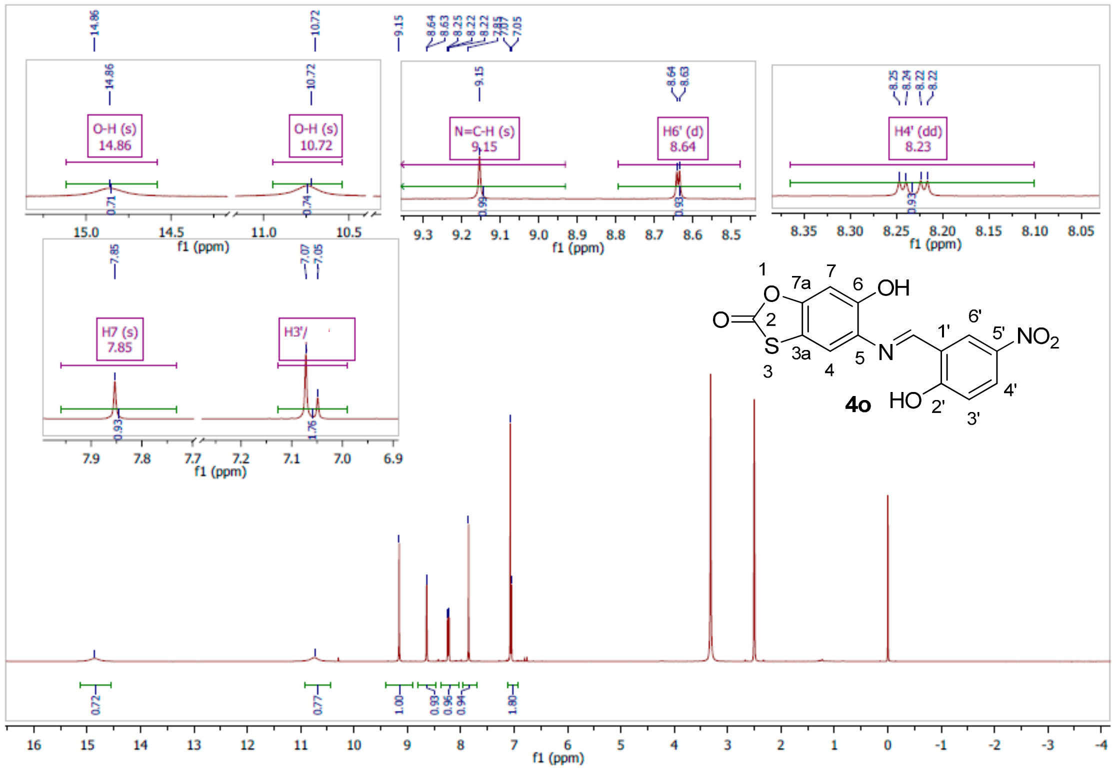

Figure S17. (*E*)-6-Hydroxy-5-(2-hydroxy-5-nitrobenzylideneamino)benzo[*d*][1,3]oxathiol-2-one (**4o**).

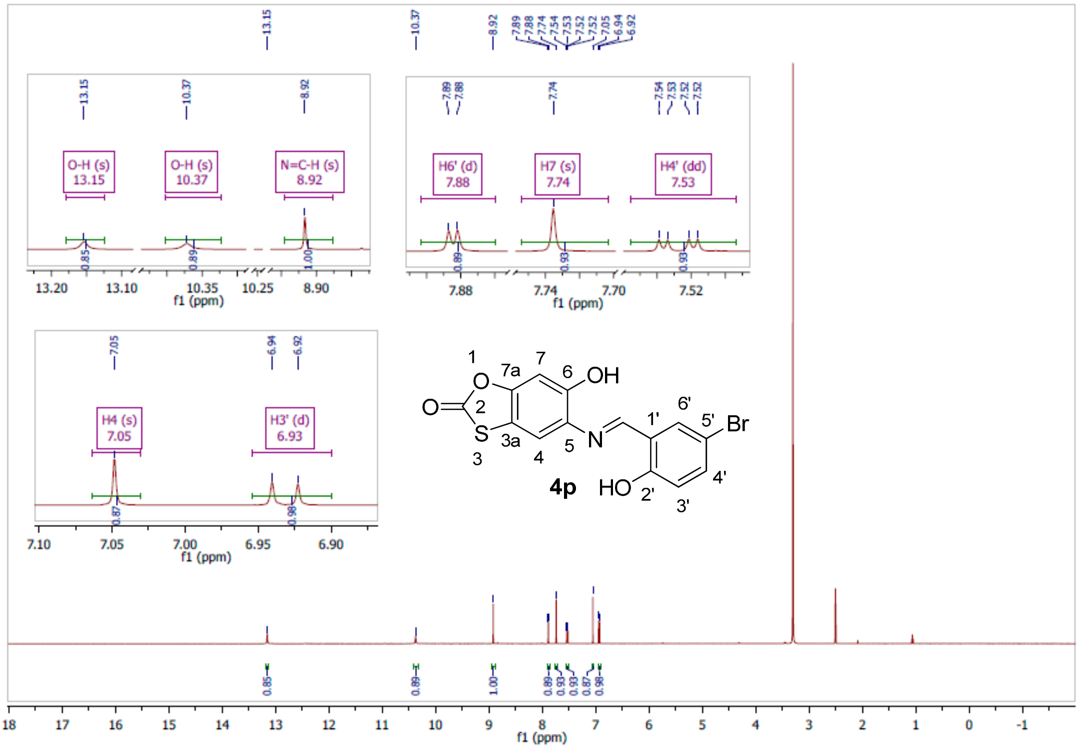

Figure S18. (*E*)-5-(5-Bromo-2-hydroxybenzylideneamino)-6-hydroxybenzo[*d*][1,3]oxathiol-2-one (**4p**).

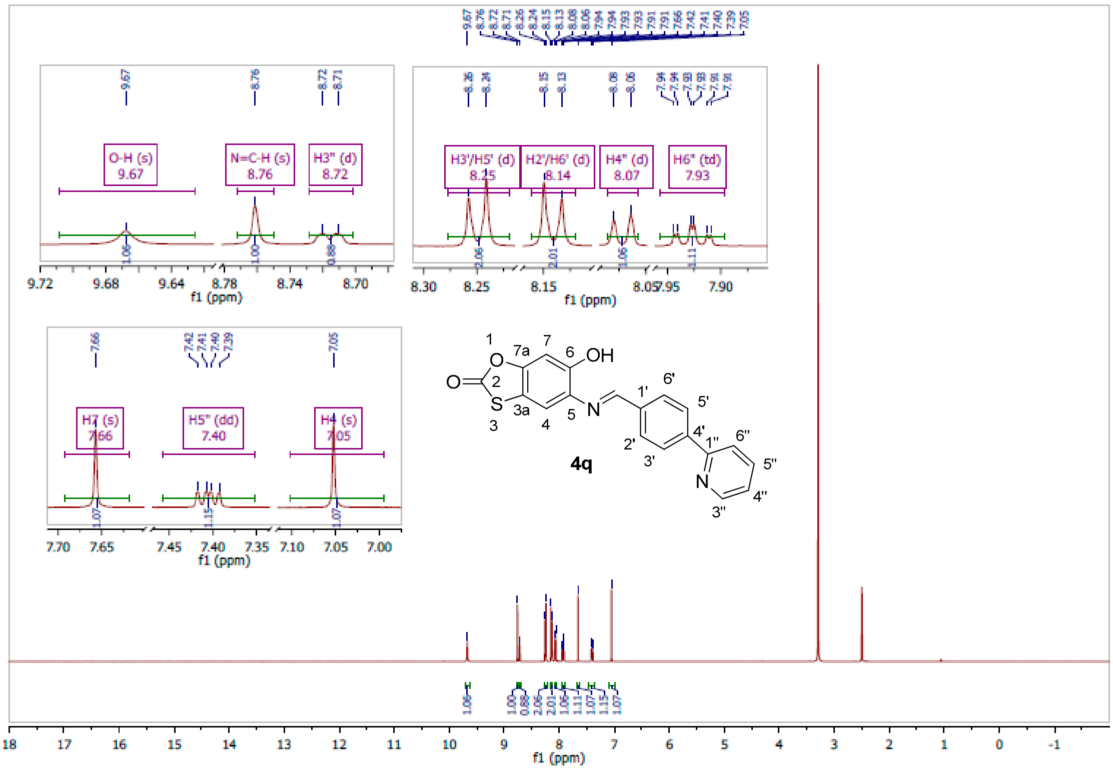

Figure S19. (*E*)-6-Hydroxy-5-(4-(pyridin-2-yl)benzylideneamino)benzo[*d*][1,3]oxathiol-2-one (**4q**).

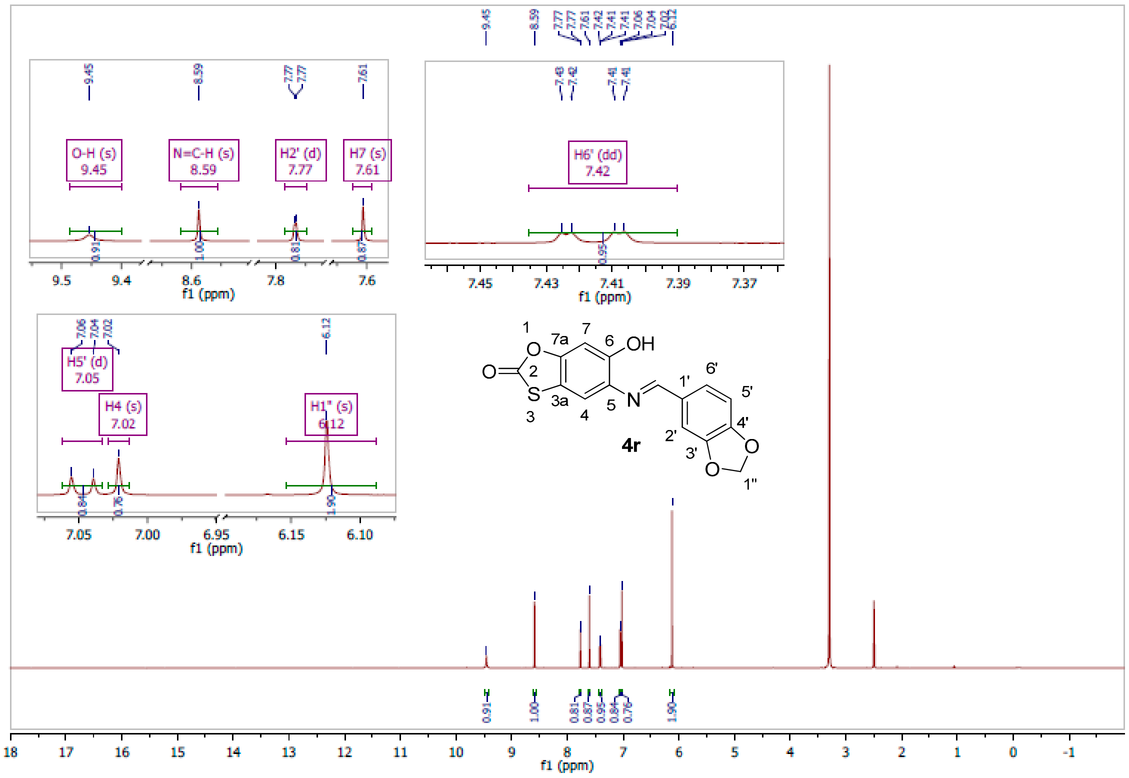

Figure S20. (*E*)-5-(Benzo[*d*][1,3]dioxol-5-ylmethyleneamino)-6-hydroxybenzo[*d*][1,3]oxathiol-2-one (**4r**).
